# A Comprehensive Multiomics Analysis Identified Ubiquilin 4 as a Promising Prognostic Biomarker of Immune-Related Therapy in Pan-Cancer

**DOI:** 10.1155/2021/7404927

**Published:** 2021-09-07

**Authors:** Jie Li, Ye Tong, Zhi Wang, Yi Liu, Xiaobo Dai, Yuxi Zhu

**Affiliations:** ^1^Department of Oncology, The First Affiliated Hospital of Chongqing Medical University, Chongqing 400016, China; ^2^Department of Oncology, Jinshan Hospital of the First Affiliated Hospital of Chongqing Medical University, Chongqing 400016, China; ^3^Chongqing Clinical Cancer Research Center, The First Affiliated Hospital of Chongqing Medical University, Chongqing 400016, China; ^4^Department of Orthopedic Surgery, Suzhou Hospital of Anhui Medical University, Suzhou, Anhui 233000, China

## Abstract

Recently, it was reported that ubiquilin 4 (UBQLN4) alteration was associated with genomic instability in some cancers. However, whether UBQLN4 is a valuable biomarker for the prognosis of immunotherapy in pan-cancer was not identified. We evaluated the biologic and oncologic significance of UBQLN4 in pan-cancer at multiomics level, such as expression, mutation, copy number variation (CNV), methylation, and N^6^-methyladenosine (m^6^A) methylation. These omics data were obtained from several public databases, including Oncomine, The Cancer Genome Atlas (TCGA), Gene Expression Omnibus (GEO), the Genotype-Tissue Expression (GTEx), the Human Protein Atlas (HPA), Gene Set Cancer Analysis (GSCA), m^6^A-Atlas, CancerSEA, and RNAactDrug. We found that UBQLN4 mRNA and protein were overexpressed in most cancer types, and the expression, mutation, CNV, and methylation of UBQLN4 were associated with the prognosis of some cancers. Mechanistically, UBQLN4 was involved in angiogenesis, DNA damage, apoptosis, and the pathway of PI3K/AKT and TSC/mTOR. Moreover, UBQLN4 mRNA was significantly correlated with immune checkpoints, tumor mutational burden (TMB), microsatellite instability (MSI), and mismatch repair (MMR). And, the correlation among UBQLN4 mRNA, CNV, and methylation and immune microenvironment was also identified. Furthermore, UBQLN4 was associated with the sensitivity of chemotherapy and targeted drugs at multiomics level. In conclusion, UBQLN4 was a promising prognostic biomarker of immune-related therapy in pan-cancer.

## 1. Introduction

Ubiquilin 4 (UBQLN4) is a member of UBQLNs family, which could bind proteasome subunits and ubiquitin proteins to degrade proteasome through its ubiquitin-like (UBL) and ubiquitin-associated (UBA) domains, respectively [1]. In the past, studies primarily focused on the role of UBQLN4 in maintaining protein homeostasis [2, 3]. Recently, it was reported that the alteration of UBQLN4 resulted in genomic instability through affecting DNA damage repair [4], which is one of the most important features of cancers [5].

Gradually, the role of UBQLN4 in cancers attracted increasing attention [6, 7], although theories behind are premature. It was reported that the increase of UBQLN4 in neuroblastoma and melanoma decreased survival [4]. Yan Yu et al. found that UBQLN4 promoted the proliferation and invasion of hepatocellular carcinoma cells by activating wnt-*β*-catenin pathway [8]. On the contrary, Shengkai Huang et al. revealed that the overexpression of UBQLN4 induced G1/S cell cycle arrest and activated p53/p21 axis, thus inhibiting the proliferation of gastric cancer cells [9]. In addition, the overexpression of UBQLN4 was reported to upregulate the sensitivity of PARP1 inhibitors [4]. Regrettably, the role of UBQLN4 in pan-cancer remains unknown.

The immunotherapy has dramatically changed the clinical practice in recent decade. However, the efficacy of immunotherapy is still needed to be improved, and it is better to find the population who is sensitive to immunotherapy before treatment. In this study, we performed comprehensive analyses of UBQLN4 in pan-cancer based on multiomics data, and UBQLN4 was identified as a promising immune and prognostic molecular biomarker. Furthermore, the role of UBQLN4 in immunotherapy and drug sensitivity might shed light on future oncotherapy.

## 2. Materials and Methods

### 2.1. Downloaded Data

We downloaded the latest data of The Cancer Genome Atlas (TCGA) of UBQLN4 RNA-sequencing, somatic mutation, and related clinical data in 33 types of cancer via UCSC Xena platform (http://xena.ucsc.edu) [10]. The clinical data included overall survival (OS), disease-specific survival (DSS), disease-free interval (DFI), and progression-free interval (PFI) data of patients with 33 cancers. The detailed abbreviations of 33 cancer types are included in Supplementary Table 1.

### 2.2. UBQLN4 mRNA Expression Profile in Pan-Cancer

To estimate the differential expression of UBQLN4 mRNA between tumor samples and normal samples in pan-cancer, Oncomine, TCGA, and the Genotype-Tissue Expression (GTEx) databases were utilized. The Oncomine database (http://www.oncomine.com) [11], which provides comprehensive analyses for cancer transcriptome data across over 18,000 gene expression microarrays, was utilized for UBQLN4 mRNA differential expression analysis in different cancers, and the threshold was set as *P* < 0.05 and fold change > 2. Tumor Immune Estimation Resource, version 2 (TIMER2.0; http://timer.cistrome.org/) [12], platform enables users to explore various cancer-related analyses based on TCGA database, including gene differential expression analysis and coexpression analysis. The “Gene_DE” module in TIMER2.0 was obtained for UBQLN4 mRNA differential expression analysis among TCGA tumors, and the Wilcoxon test was applied for statistical significance. However, the lack of normal samples in several cancers in TCGA database limited the integrated analysis. Gene Expression Profiling Interactive Analysis, version 2 (GEPIA2; http://gepia.cancer-pku.cn/) [13], web server has integrated tumor and normal tissue samples of TCGA and GTEx databases and was applied for differential analysis of UBQLN4 mRNA in adrenocortical carcinoma (ACC), lymphoid neoplasm diffuse large B-cell lymphoma (DLBC), acute myeloid leukemia (LAML), brain lower grade glioma (LGG), ovarian serous cystadenocarcinoma (OV), sarcoma (SARC), testicular germ cell tumor (TGCT), thymoma (THYM), and uterine carcinosarcoma (UCS). |Log2FC| > 1 and *P* value <0.05 was set as the cutoff.

### 2.3. UBQLN4 mRNA Expression and Clinical Prognosis

The correlation between UBQLN4 mRNA expression and clinical prognosis in different cancer types was analyzed using R software (v.3.6.3) [14]. Primarily, the OS, DSS, DFI, and PFI analyses in every cancer were calculated, and Kaplan–Meier curves were drawn using “survival” and “survminer” packages. Furthermore, the cox regression analyses were performed using “survival” and “forestplot” packages. The cutoff value was set as the median level of UBQLN4. *P* < 0.05 was thought as significant and would be visualized.

PrognoScan database (http://www.prognoscan.org) [15] collected gene expression and clinical data from Gene Expression Omnibus (GEO), ArrayExpress, and individual laboratory websites. And, we verified the prognostic value of UBQLN4 in different cancers using PrognoScan database.

### 2.4. UBQLN4 Protein Expression Profile

The Human Protein Atlas (HPA) and Clinical Proteomic Tumor Analysis Consortium (CPTAC) databases were utilized for UBQLN4 protein analysis. HPA portal (http://www.proteinatlas.org) [16] offers the human proteome map in various tissues, and it was used for the immunofluorescence and the immunohistochemistry analyses of UBQLN4 protein. UALCAN (http://ualcan.path.uab.edu) [17] is a web resource which facilitates protein differential analysis using CPTAC database for different tumor types. We compared the UBQLN4 protein expression level between tumor tissues and normal tissues in breast invasive carcinoma (BRCA), lung adenocarcinoma (LUAD), colon adenocarcinoma (COAD), and OV using UALCAN and HPA.

### 2.5. UBQLN4 Mutation Profile in Pan-Cancer

cBioPortal (http://www.cbioportal.org/) [18] is an integrated web resource which can be exploited for genomic alterations analyses for a specific gene, including OncoPrint tab, Cancer Types Summery tab, Mutations tab, and Survival tab. In this study, we applied the OncoPrint tab for visualizing genomic alterations of UBQLN4 among 32 cancer types of TCGA. Simultaneously, the alteration frequency in every cancer type was plotted by Cancer Types Summery tab. Furthermore, protein domains and the positions of specific mutations for UBQLN4 were illustrated based on the Mutations tab. To investigate the prognostic value of UBQLN4 mutation, Survival tab was also applied.

### 2.6. UBQLN4 CNV Profile in Pan-Cancer Based on GSCA

Gene Set Cancer Analysis (GSCA; http://bioinfo.life.hust.edu.cn/web/GSCA/) [19] platform is a web server that integrated multiomics data based on TCGA database. GSCA provides several analyses, including copy number variation (CNV), methylation, pathway activity, and immune infiltrates. Based on CNV module of GSCA, the proportion of UBQLN4 heterozygous/homozygous and amplification/deletion, Spearman correlation between UBQLN4 mRNA expression and CNV, and the survival difference between UBQLN4 CNV and wild type were displayed in pan-cancer.

### 2.7. UBQLN4 Methylation Profile in Pan-Cancer Based on GSCA

The Methylation module in GSCA was used to estimate the UBQLN4 differential methylation between tumor and normal samples, Spearman correlation between UBQLN4 mRNA expression and methylation, and OS difference between UBQLN4 hypermethylation and hypomethylation in different cancer types. Furthermore, the “Gene_Corr” module in TIMER2.0 was used to investigate the coexpression pattern between UBQLN4 and known transmethylase-related genes (DNMT1, DNMT3A, and DNMT3B) in pan-cancer, and Spearman correlation analysis was performed.

### 2.8. m^6^A Methylation Analysis of UBQLN4 Based on m^6^A-Atlas

The N^6^-methyladenosine (m^6^A)-Atlas (http://www.xjtlu.edu.cn/biologicalsciences/atlas) [20] is an interactive platform that facilitates comprehensive m^6^A epitranscriptome analyses based on GEO database and Genome Sequence Archive in BIG Data Center. We identified all the m^6^A records on UBQLN4 and chose one of these records (Human_m6A_14897) for further analysis. Different RNA modification sites distribution in UBQLN4 and the detailed site of Human_m6A_14897 were illustrated. Moreover, the m^6^A methylation levels of Human_m6A_14897 under different conditions and comethylated genes network of Human_m6A_14897 were also displayed. And the RNA Binding Region, miRNA-Targets, predicted gene ontology (GO) functions, and comethylated m^6^A sites for UBQLN4 were also analyzed by m^6^A-Atlas.

### 2.9. Pathway Exploration for UBQLN4 in Pan-Cancer Based on Different Databases

To further understand the functions and pathways of UBQLN4 in pan-cancer, different databases were searched. CancerSEA database (http://biocc.hrbmu.edu.cn/CancerSEA/) [21] firstly provides distinct functional states of specific genes in different cancer types at the single-cell level which could avoid the limitation of tumor heterogeneity. The correlations between the UBQLN4 and functional states in various cancers were performed based on CancerSEA database. Furthermore, the Pathway Activity module in GSCA was used to present the effect of UBQLN4 on cancer-related pathways (activation or inhibition) in pan-cancer.

### 2.10. Correlation and Functional Analyses between UBQLN4 and Other Members in UBQLNs Family

Since the high degree of similarity among UBQLNs family, we explored the correlation and function between UBQLN4 and other members in UBQLNs family (UBQLN1, UBQLN2, UBQLN3, and UBQLNL). “Gene_Corr” module in TIMER2.0 was used to investigate the coexpression pattern between UBQLN4 and other members in UBQLNs family in pan-cancer, and Spearman correlation analysis was performed. Furthermore, the Pathway Activity module in GSCA was used to present the effect of UBQLNs family on cancer-related pathways (activation or inhibition) in pan-cancer.

### 2.11. Association between UBQLN4 mRNA Expression and Immunotherapy and Immune Microenvironment in Pan-Cancer Based on TCGA Database

R software (v.3.6.3) was utilized for the association between UBQLN4 mRNA expression and immunotherapy and immune microenvironment. The coexpression analyses of UBQLN4 mRNA and immune checkpoints and mismatch repair (MMR) signatures in pan-cancer were performed using “limma” package based on TCGA database, and Pearson test was performed. Furthermore, “reshape2” and “RColorBrewer” packages were used to visualize these heatmaps.

Tumor mutational burden (TMB) was the number of mutated bases per megabase, which was effective for evaluating the response to immunotherapy [22]. Furthermore, microsatellite instability (MSI) status was also a biomarker in selecting the dominant group of immunotherapy [23]. In this study, Spearman correlation analysis was applied into the association between UBQLN4 mRNA expression and TMB and MSI based on the somatic mutation data from TCGA database, and “fmsb” package was used to visualize these results.

Furthermore, to explore the correlation between UBQLN4 mRNA and immune microenvironment, immune score and stromal score were calculated using “estimate” and “limma” packages. CIBERSORT [24] was used to evaluate the relationship between UBQLN4 mRNA and immune cells infiltration. And “ggplot2,” “ggpubr,” and “ggExtra” packages were used to visualize these results. Spearman test was used for the correlation coefficient calculation.

### 2.12. Correlation between UBQLN4 CNV and Methylation and Immune Microenvironment Based on GSCA Database

Immune infiltrates module in GSCA presents the Spearman correlation between immune infiltrates and CNV and methylation in pan-cancer based on Immune Cell Abundance Identifier (ImmuCellAI) tool [25]. As a result, the correlation between UBQLN4 CNV and methylation and immune infiltrates was estimated using the immune infiltrates module of GSCA.

### 2.13. Drug Sensitivity Analysis Based on Multiomics Data

RNAactDrug (http://bio-bigdata.hrbmu.edu.cn/RNAactDrug) [26] database integrated the data of Drug Sensitivity in Cancer (GDSC), CellMiner, and Cancer Cell Line Encyclopedia (CCLE) databases, which offered the analyses of correlation between drug sensitivity and RNA molecule at multiomics level. The association between drug sensitivity and UBQLN4 mRNA at expression, mutation, CNV, and methylation level was performed to further guide clinical practice. Pearson and Spearman correlation analyses were applied for the correlation between drug sensitivity data and UBQLN4 mRNA.

### 2.14. Statistical Analysis

R software (v.3.6.3) was utilized for statistical analysis in this study. The differential expression analyses between normal and tumor tissues were based on *t* tests. Kaplan–Meier curves and cox regression analyses were performed to identify the correlation between high and low UBQLN4 expression levels and OS, DSS, DFI, and PFI in pan-cancer. And the cutoff value was set as the median level of UBQLN4. Furthermore, the correlation analyses between UBQLN4 and other variables were analyzed by Spearman or Pearson test. And *P* value <0.05 was considered as statistically significant.

## 3. Results

### 3.1. UBQLN4 mRNA Expression Profile in Pan-Cancer Based on Different Databases

**Figure 1:**
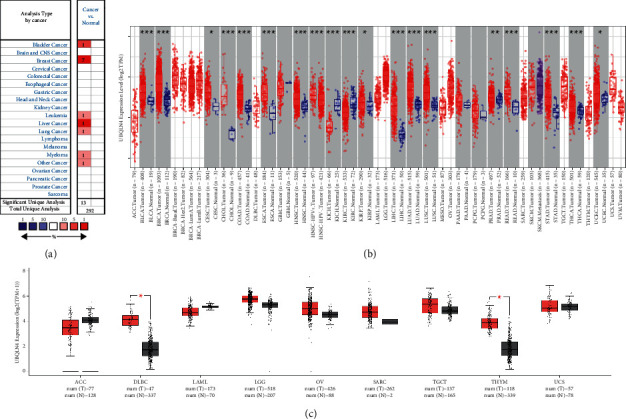
The differential mRNA expression analyses of UBQLN4 in pan-cancer based on Oncomine, TCGA, and GTEx databases. (a) The differential expression analysis of UBQLN4 mRNA in various cancers based on Oncomine database. Red represents upregulation of UBQLN4 mRNA and the number stands for the count of the significant unique analyses (*P* < 0.05). (b) The differential expression analysis of UBQLN4 mRNA between cancers and normal tissues using TIMER2.0 based on TCGA database; ^*∗*^*P* < 0.05, ^*∗∗*^*P* < 0.01, and ^*∗∗∗*^*P* < 0.001. (c) The differential expression analysis of UBQLN4 mRNA in different cancers using GEPIA based on TCGA and GTEx databases. Red represents tumor tissues and grey represents normal tissues; ^*∗*^*P* < 0.05. UBQLN4: ubiquilin 4; TCGA: The Cancer Genome Atlas; GTEx: Genotype-Tissue Expression Project; TIMER2.0: Tumor Immune Estimation Resource, version 2; GEPIA: Gene Expression Profiling Interactive Analysis; ACC: adrenocortical carcinoma; BLCA: bladder urothelial carcinoma; BRCA: breast invasive carcinoma; CESC: cervical squamous cell carcinoma and endocervical adenocarcinoma; CHOL: cholangiocarcinoma; COAD: colon adenocarcinoma; DLBC: lymphoid neoplasm diffuse large B-cell lymphoma; ESCA: esophageal carcinoma; GBM: glioblastoma multiforme; HNSC: head and neck squamous cell carcinoma; KICH: kidney chromophobe; KIRC: kidney renal clear cell carcinoma; KIRP: kidney renal papillary cell carcinoma; LAML: acute myeloid leukemia; LGG: brain lower grade glioma; LIHC: liver hepatocellular carcinoma; LUAD: lung adenocarcinoma; LUSC: lung squamous cell carcinoma; MESO: mesothelioma; OV: ovarian serous cystadenocarcinoma; PAAD: pancreatic adenocarcinoma; PCPG: pheochromocytoma and paraganglioma; PRAD: prostate adenocarcinoma; READ: rectum adenocarcinoma; SARC: sarcoma; SKCM: skin cutaneous melanoma; STAD, stomach adenocarcinoma; TGCT: testicular germ cell tumor; THCA: thyroid carcinoma; THYM: thymoma; UCEC: uterine corpus endometrial carcinoma; UCS: uterine carcinosarcoma; UVM: uveal melanoma.

We utilized Oncomine, TCGA, and GTEx databases for differential expression analysis of UBQLN4 mRNA in various cancer types. As shown in Figure 1(a), UBQLN4 mRNA was upregulated in bladder cancer, breast cancer, leukemia, liver cancer, lung cancer, and myeloma based on Oncomine database (*P* < 0.05). According to TCGA database, upregulation of UBQLN4 mRNA was observed in bladder urothelial carcinoma (BLCA), BRCA, cervical squamous cell carcinoma and endocervical adenocarcinoma (CESC), cholangiocarcinoma (CHOL), COAD, esophageal carcinoma (ESCA), head and neck squamous cell carcinoma (HNSC), kidney renal papillary cell carcinoma (KIRP), liver hepatocellular carcinoma (LIHC), LUAD, lung squamous cell carcinoma (LUSC), prostate adenocarcinoma (PRAD), rectum adenocarcinoma (READ), stomach adenocarcinoma (STAD), thyroid carcinoma (THCA), and uterine corpus endometrial carcinoma (UCEC), while UBQLN4 mRNA was downregulated in kidney chromophobe (KICH) and kidney renal clear cell carcinoma (KIRC) (Figure 1(b)). As for the lack of normal tissues data of some cancers in TCGA database, GEPIA2 was utilized for further analysis. After integrating tumor and normal tissue samples of TCGA and GTEx databases, UBQLN4 mRNA was significantly upregulated in DLBC and THYM compared with normal tissues (*P* < 0.05; Figure 1(c)).

### 3.2. Survival Analysis of UBQLN4 mRNA in Pan-Cancer Based on Different Databases

**Figure 2:**
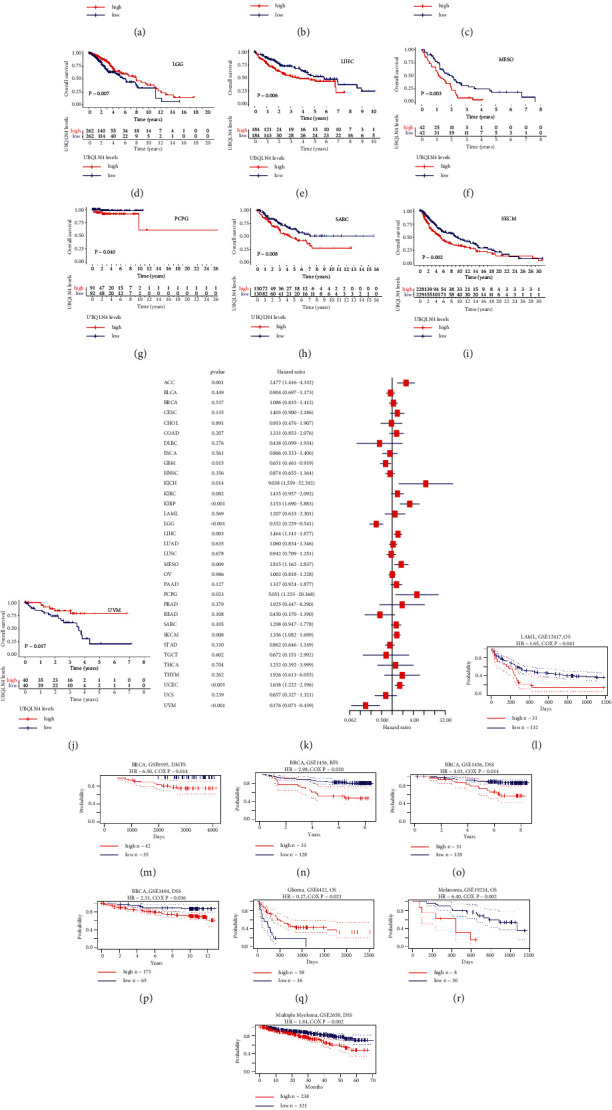
The survival analysis of UBQLN4 mRNA in pan-cancer based on TCGA and GEO database in pan-cancer. OS curves of UBQLN4 in ACC (a), GBM (b), KICH (c), LGG (d), LIHC (e), MESO (f), PCPG (g), SARC (h), SKCM (i), and UVM (j) with significance based on TCGA database. (k) The cox regression analysis for OS and UBQLN4 expression in 33 cancer types based on TCGA database. The Kaplan–Meier curves of UBQLN4 in LAML (l), BRCA (M-P), glioma (q), melanoma (r), and multiple myeloma (s) with significance based on GEO database. UBQLN4: ubiquilin 4; TCGA: The Cancer Genome Atlas; GEO: Gene Expression Omnibus; OS: overall survival; ACC: adrenocortical carcinoma; BLCA: bladder urothelial carcinoma; BRCA: breast invasive carcinoma; CESC: cervical squamous cell carcinoma and endocervical adenocarcinoma; CHOL: cholangiocarcinoma; COAD: colon adenocarcinoma; DLBC: lymphoid neoplasm diffuse large B-cell lymphoma; ESCA: esophageal carcinoma; GBM: glioblastoma multiforme; HNSC: head and neck squamous cell carcinoma; KICH: kidney chromophobe; KIRC: kidney renal clear cell carcinoma; KIRP: kidney renal papillary cell carcinoma; LAML: acute myeloid leukemia; LGG: brain lower grade glioma; LIHC: liver hepatocellular carcinoma; LUAD: lung adenocarcinoma; LUSC: lung squamous cell carcinoma; MESO: mesothelioma; OV: ovarian serous cystadenocarcinoma; PAAD: pancreatic adenocarcinoma; PCPG: pheochromocytoma and paraganglioma; PRAD: prostate adenocarcinoma; READ: rectum adenocarcinoma; SARC: sarcoma; SKCM: skin cutaneous melanoma; STAD, stomach adenocarcinoma; TGCT: testicular germ cell tumor; THCA: thyroid carcinoma; THYM: thymoma; UCEC: uterine corpus endometrial carcinoma; UCS: uterine carcinosarcoma; UVM: uveal melanoma.

To evaluate the prognostic value of UBQLN4 mRNA in pan-cancer, OS, DSS, DFI, and PFI analysis were performed based on TCGA database. OS curves indicated that high expression level of UBQLN4 mRNA was associated with poor prognosis in ACC, KICH, LIHC, mesothelioma (MESO), pheochromocytoma and paraganglioma (PCPG), SARC, and skin cutaneous melanoma (SKCM), while low expression level of UBQLN4 mRNA was associated with poor prognosis in glioblastoma multiforme (GBM), LGG, and uveal melanoma (UVM) (Figures 2(a)-2(j)). The cox regression analysis revealed that lower expression of UBQLN4 mRNA was correlated with longer OS in ACC, KICH, KIRP, LIHC, MESO, PCPG, SKCM, and UCEC, but it is correlated with shorter OS in GBM, LGG, and UVM (Figure 2(k)). Furthermore, DSS curves in Supplementary Figures 1A–1J suggested that high expression level of UBQLN4 mRNA was associated with poor prognosis in ACC, KICH, MESO, PCPG, PRAD, SARC, SKCM, and UVM, and low expression level of UBQLN4 mRNA was associated with poor prognosis in GBM and LGG. The cox regression analysis revealed that lower expression of UBQLN4 mRNA was a protective factor in ACC, KIRP, MESO, PCPG, SKCM, and UCEC, but it is a risk factor in GBM, LGG, and UVM (Supplementary Figure 1K). As illustrated in Supplementary Figures 2A–2C, DFI curves indicated that low expression level of UBQLN4 mRNA was associated with better prognosis in READ, SARC, and UCEC. The cox regression analysis revealed that lower expression of UBQLN4 mRNA was a protective factor in LIHC, PRAD, UCEC, and UCS, but it is a risk factor in ESCA (Supplementary Figure 2D). PFI curves indicated that high expression level of UBQLN4 mRNA was associated with poor prognosis in ACC, LIHC, MESO, SARC, THYM, and UCEC, while low expression level of UBQLN4 mRNA was associated with poor prognosis in GBM and LGG (Supplementary Figures 3A–H). The cox regression analysis revealed that lower expression of UBQLN4 mRNA was a protective factor in ACC, LIHC, MESO, PCPG, PRAD, THCA, THYM, and UCEC, but it is a risk factor in GBM, LGG, and UVM (Supplementary Figure 3I).

For further identifying the prognostic significance of UBQLN4 mRNA, GEO database was also utilized. Low expression of UBQLN4 mRNA was correlated with longer OS in LAML and melanoma; longer distant metastasis-free survival (DMFS), progression-free survival (PFS), and DSS in BRCA; longer DSS in multiple myeloma; and shorter OS in glioma according to GEO database (Figures 2(l)–2(s)) [27–33].

### 3.3. UBQLN4 Protein Expression Profile Based on Different Databases

**Figure 3:**
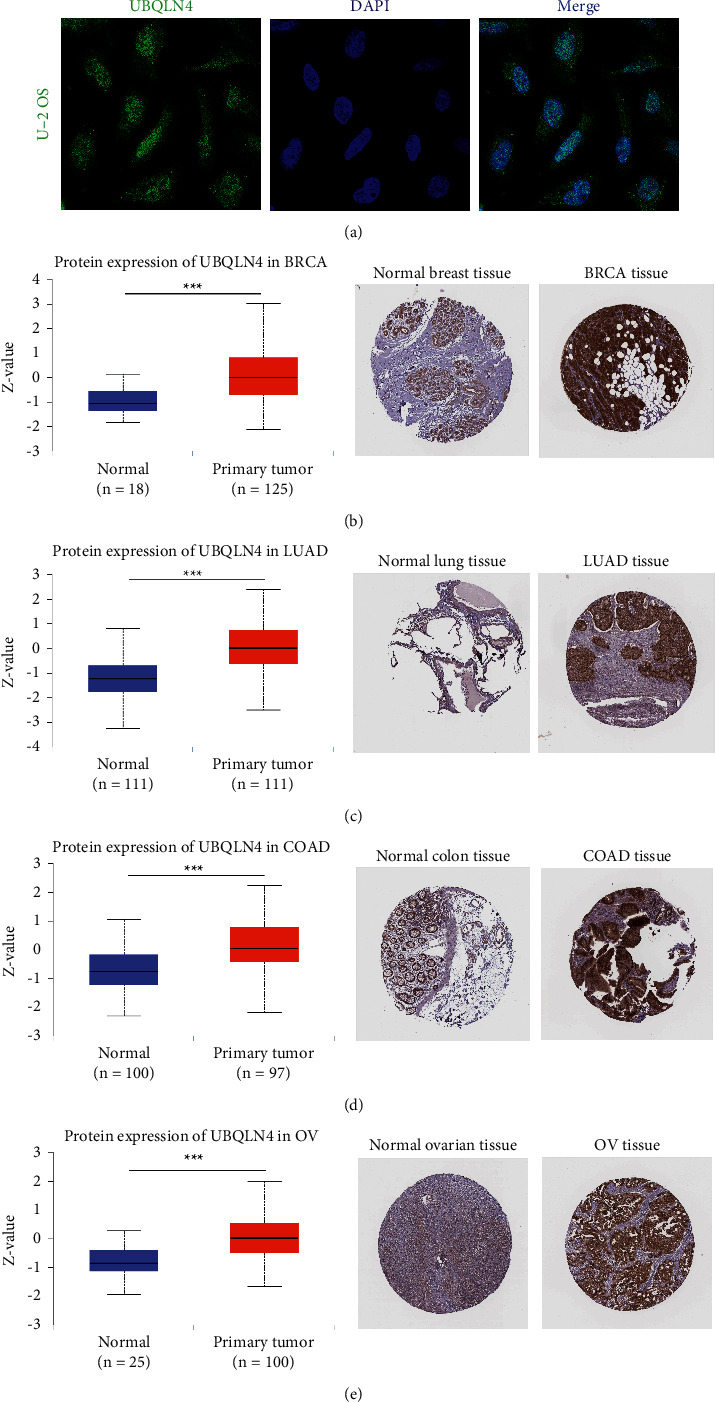
The differential protein expression analyses of UBQLN4 in pan-cancer based on HPA and CPTAC database. (a) UBQLN4 protein is almost located in the nucleoplasm in human sarcoma U-2 OS cell line based on the immunofluorescence analysis from HPA database. The differential protein expression and immunohistochemistry analyses of UBQLN4 in BRCA (B), LUAD (c), COAD (d), and OV (e) based on CPTAC and HPA database. UBQLN4: ubiquilin 4; HPA: The Human Protein Atlas; CPTAC: Clinical Proteomic Tumor Analysis Consortium; BRCA: breast invasive carcinoma; LUAD: lung adenocarcinoma; COAD: colon adenocarcinoma; OV: ovarian serous cystadenocarcinoma.

We obtained the subcellular location of UBQLN4 protein by HPA database. As illustrated in Figure 3(a), UBQLN4 protein was almost located in the nucleoplasm based on the immunofluorescence analysis in human sarcoma U-2 OS cell line. Furthermore, UBQLN4 protein was significantly overexpressed in tumor tissues compared with normal tissues in BRCA, LUAD, COAD, and OV based on the differential protein expression analysis in CPTAC database (Figures 3(b)–3(e)). Furthermore, the immunohistochemistry analyses of UBQLN4 protein in HPA database confirmed the overexpression of UBQLN4 protein in these cancers.

### 3.4. UBQLN4 Mutation Profile in Pan-Cancer Based on cBioPortal

**Figure 4:**
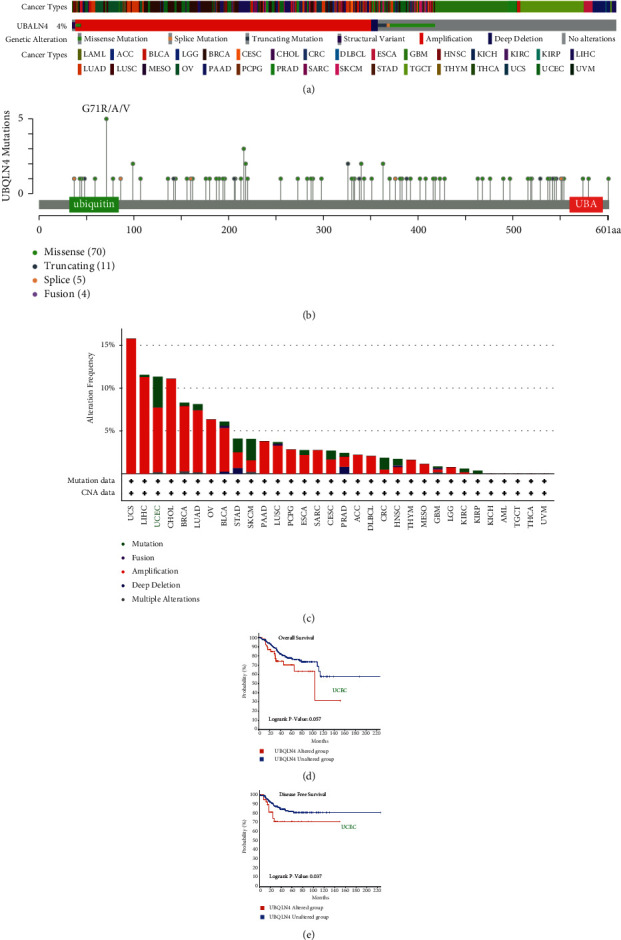
The mutation landscape of UBQLN4 in pan-cancer based on cBioPortal. UBQLN4 alteration types (a), mutation sites (b), and alteration frequency (c) in different cancer types. The OS and DFS analysis between UBQLN4 altered group and unaltered group in UCEC. UBQLN4: ubiquilin 4; OS: overall survival; DFS: disease-free survival; ACC: adrenocortical carcinoma; BLCA: bladder urothelial carcinoma; BRCA: breast invasive carcinoma; CESC: cervical squamous cell carcinoma and endocervical adenocarcinoma; CHOL: cholangiocarcinoma; CRC: colorectal carcinoma; DLBCL: diffuse large B-cell lymphoma; ESCA: esophageal carcinoma; GBM: glioblastoma multiforme; HNSC: head and neck squamous cell carcinoma; KICH: kidney chromophobe; KIRC: kidney renal clear cell carcinoma; KIRP: kidney renal papillary cell carcinoma; LAML: acute myeloid leukemia; LGG: brain lower grade glioma; LIHC: liver hepatocellular carcinoma; LUAD: lung adenocarcinoma; LUSC: lung squamous cell carcinoma; MESO: mesothelioma; OV: ovarian serous cystadenocarcinoma; PAAD: pancreatic adenocarcinoma; PCPG: pheochromocytoma and paraganglioma; PRAD: prostate adenocarcinoma; SARC: sarcoma; SKCM: skin cutaneous melanoma; STAD, stomach adenocarcinoma; TGCT: testicular germ cell tumor; THCA: thyroid carcinoma; THYM: thymoma; UCEC: uterine corpus endometrial carcinoma; UCS: uterine carcinosarcoma; UVM: uveal melanoma.

The mutation landscape of UBQLN4 was analyzed using cBioPortal based on TCGA database. As shown in Figure 4(a), total alteration frequency of UBQLN4 was 4% among 32 cancer types of TCGA. Furthermore, the detailed mutation sites are illustrated in Figure 4(b). 90 mutation sites (including 70 missense, 11 truncating, 5 splice, and 4 fusion mutations) were found in UBQLN4, which were located between amino acids 0 and 601, and G71 R/A/V had the highest mutation frequency. Furthermore, the alteration profile in various cancer types is also shown in Figure 4(c). Among 32 cancer types, UCS had the highest amplification alteration frequency (>15%), and UCEC had the highest mutation frequency (>3%). To further explore the prognostic value of UBQLN4 alteration, we performed survival analysis in UCEC. The results indicated that UBQLN4 unaltered group had better OS and disease-free survival (DFS) than UBQLN4 altered group (Figures 4(d)–4(e)).

### 3.5. UBQLN4 CNV Profile in Pan-Cancer Based on GSCA

**Figure 5:**
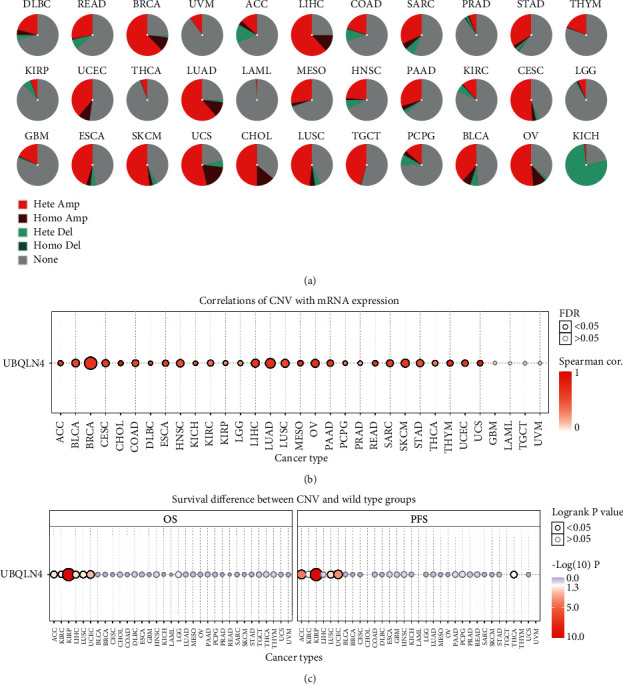
The CNV landscape of UBQLN4 in pan-cancer based on GSCA. (a) The deletion/amplification of heterozygous/homozygous CNV for UBQLN4 in various cancer types. (b) Correlation between CNV and UBQLN4 mRNA expression in various cancers. (c) Survival difference between CNV and wide type groups in pan-cancer. CNV: copy number variation; UBQLN4: ubiquilin 4; GSCA: Gene Set Cancer Analysis; Hete Amp: Heterozygous Amplification; Homo Amp: Homozygous Amplification; Hete Del: Heterozygous Deletion; Homo Del: Homozygous Deletion; OS: overall survival; PFS: progression-free survival; ACC: adrenocortical carcinoma; BLCA: bladder urothelial carcinoma; BRCA: breast invasive carcinoma; CESC: cervical squamous cell carcinoma and endocervical adenocarcinoma; CHOL: cholangiocarcinoma; COAD: colon adenocarcinoma; DLBC: lymphoid neoplasm diffuse large B-cell lymphoma; ESCA: esophageal carcinoma; GBM: glioblastoma multiforme; HNSC: head and neck squamous cell carcinoma; KICH: kidney chromophobe; KIRC: kidney renal clear cell carcinoma; KIRP: kidney renal papillary cell carcinoma; LAML: acute myeloid leukemia; LGG: brain lower grade glioma; LIHC: liver hepatocellular carcinoma; LUAD: lung adenocarcinoma; LUSC: lung squamous cell carcinoma; MESO: mesothelioma; OV: ovarian serous cystadenocarcinoma; PAAD: pancreatic adenocarcinoma; PCPG: pheochromocytoma and paraganglioma; PRAD: prostate adenocarcinoma; READ: rectum adenocarcinoma; SARC: sarcoma; SKCM: skin cutaneous melanoma; STAD, stomach adenocarcinoma; TGCT: testicular germ cell tumor; THCA: thyroid carcinoma; THYM: thymoma; UCEC: uterine corpus endometrial carcinoma; UCS: uterine carcinosarcoma; UVM: uveal melanoma.

The UBQLN4 CNV landscape in 33 cancer types was performed by GSCA database. As illustrated in Figure 5(a), a relatively higher heterozygous amplification ratio (>50%) was found in BRCA, CESC, SKCM, LIHC, LUAD, OV, and UCS, the highest heterozygous deletion ratio (>77%) was found in KICH, and the highest homozygous amplification (>19%) was found in UCS. The detailed ratio of CNV types in every cancer was shown in Supplementary Table 2. Moreover, except for UVM, TGCT, GBM, and LAML, the rest 29 cancer types were statistically significant for the correlation between UBQLN4 CNV and UBQLN4 mRNA expression (Figure 5(b)), and the detailed information is shown in Supplementary Table 3. Subsequently, the prognostic significance of UBQLN4 CNV was also analyzed in pan-cancer using GSCA database. The results suggested that UBQLN4 wild type had significantly better OS than that in amplification/deletion type in KIRP, LIHC, and UCEC and better PFS of KIRP and UCEC. While UBQLN4 deletion type in ACC and THCA had better OS and PFS than wild type. Moreover, UBQLN4 amplification type had better OS and PFS than wild type in LUSC. And the detailed survival curves are illustrated in Supplementary Figure 4.

### 3.6. UBQLN4 Methylation Profile in Pan-Cancer Based on GSCA

**Figure 6:**
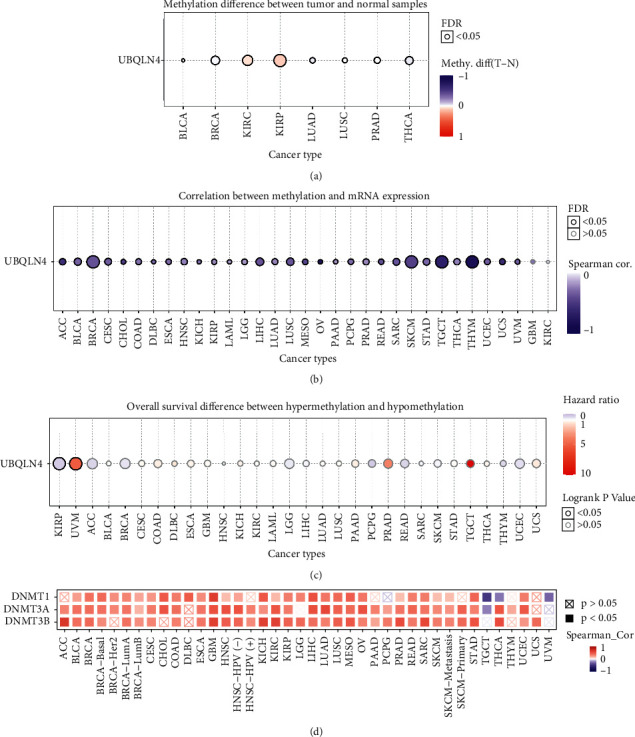
The methylation landscape of UBQLN4 in pan-cancer based on GSCA and TIMER2.0. (a) Methylation difference of UBQLN4 between tumor and normal tissues in different cancers. (b) Correlation between methylation and UBQLN4 mRNA expression. (c) Overall survival difference between high and low methylation of UBQLN4. (d) Correlation between known transmethylase genes (DNMT1, DNMT3A, and DNMT3B) and UBQLN4. UBQLN4: ubiquilin 4; GSCA: Gene Set Cancer Analysis; TIMER2.0: Tumor Immune Estimation Resource, version 2; ACC: adrenocortical carcinoma; BLCA: bladder urothelial carcinoma; BRCA: breast invasive carcinoma; CESC: cervical squamous cell carcinoma and endocervical adenocarcinoma; CHOL: cholangiocarcinoma; COAD: colon adenocarcinoma; DLBC: lymphoid neoplasm diffuse large B-cell lymphoma; ESCA: esophageal carcinoma; GBM: glioblastoma multiforme; HNSC: head and neck squamous cell carcinoma; KICH: kidney chromophobe; KIRC: kidney renal clear cell carcinoma; KIRP: kidney renal papillary cell carcinoma; LAML: acute myeloid leukemia; LGG: brain lower grade glioma; LIHC: liver hepatocellular carcinoma; LUAD: lung adenocarcinoma; LUSC: lung squamous cell carcinoma; MESO: mesothelioma; OV: ovarian serous cystadenocarcinoma; PAAD: pancreatic adenocarcinoma; PCPG: pheochromocytoma and paraganglioma; PRAD: prostate adenocarcinoma; READ: rectum adenocarcinoma; SARC: sarcoma; SKCM: skin cutaneous melanoma; STAD, stomach adenocarcinoma; TGCT: testicular germ cell tumor; THCA: thyroid carcinoma; THYM: thymoma; UCEC: uterine corpus endometrial carcinoma; UCS: uterine carcinosarcoma; UVM: uveal melanoma.

The methylation landscape of UBQLN4 in pan-cancer was also analyzed. UBQLN4 differential methylation was found in BLCA, BRCA, KIRC, KIRP, LUAD, LUSC, PRAD, and THCA compared with normal tissues (Figure 6(a)). Furthermore, UBQLN4 methylation was significantly correlated with UBQLN4 mRNA expression in most of cancer types (Figure 6(b)), and the detailed information is shown in Supplementary Table 4. Notably, prognostic significance of UBQLN4 methylation was found in KIRP and UVM (Figure 6(c)). UBQLN4 hypermethylation in KIRP and UBQLN4 hypomethylation in UVM indicated better OS (Supplementary Figure 5). Furthermore, the correlation between known transmethylase-related genes (DNMT1, DNMT3A, and DNMT3B) and UBQLN4 mRNA was also performed. The heatmap indicated that UBQLN4 mRNA was positively related to transmethylase-related genes in most cancers, and with the highest correlation score in GBM (Figure 6(d)). But UBQLN4 mRNA was negatively related to transmethylase-related genes in TGCT.

**Figure 7:**
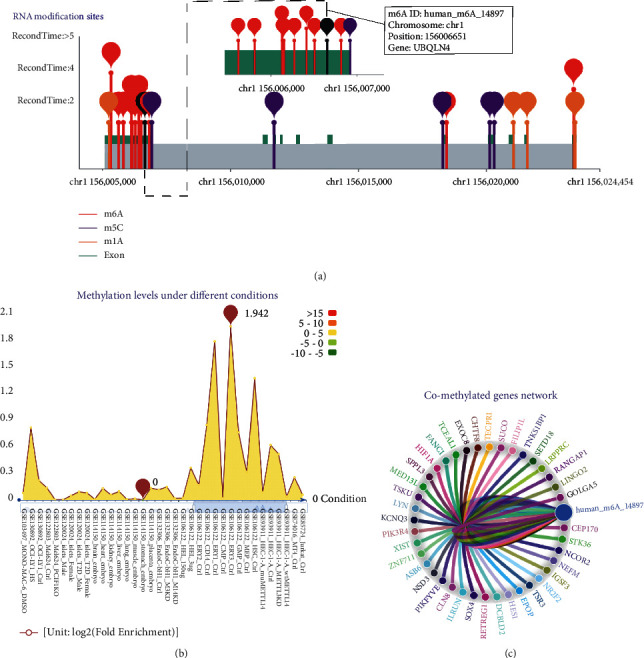
The m^6^A methylation analysis of Human_m6A_14897 based on m^6^A-Atlas. (a) Landscape of RNA modification in UBQLN4 and the detailed site of Human_m6A_14897. (b) The m^6^A methylation levels of Human_m6A_14897 under different conditions. (c) Comethylated genes network of Human_m6A_14897. m^6^A: N6-methyladenosine; UBQLN4: ubiquilin 4.

Additionally, we explored the m^6^A methylation analysis based on m^6^A-Atlas. A total of 13 m^6^A records on UBQLN4 were found based on m^6^A-Atlas (Supplementary Table 5), and Human_m6A_14897 was chosen for further analysis. The landscape of RNA modification in UBQLN4 is illustrated in Figure 7(a), and Human_m6A_14897 was located in chr1:156006651. Moreover, the m^6^A methylation levels of Human_m6A_14897 under different conditions and comethylated genes network of Human_m6A_14897 are also shown in Figure 7(b)–7(c). The detailed information of human_m6A_14897 is shown in Supplementary Table 6, including the RNA Binding Region, miRNA-Targets, predicted GO functions, and comethylated m^6^A sites.

### 3.7. Pathway Exploration for UBQLN4 in Pan-Cancer Based on Different Databases

**Figure 8:**
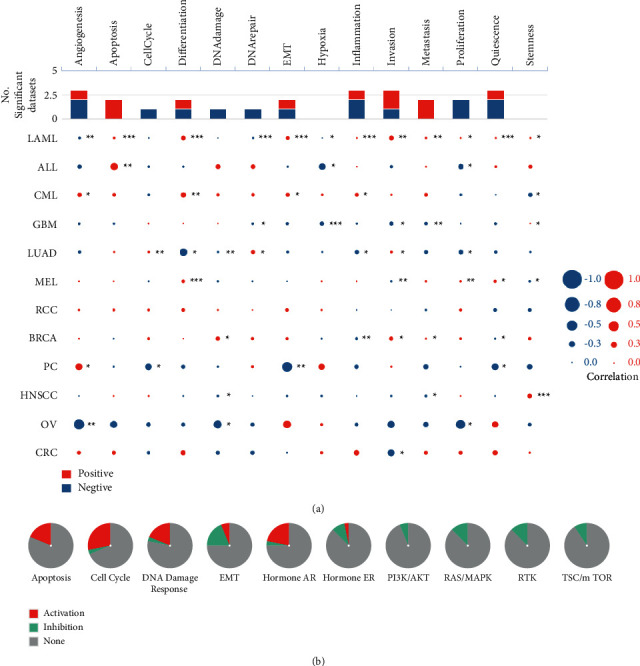
(a) The correlation between functional states and UBQLN4 in different cancers based on CancerSEA. Red stands for a positive correlation, and blue stands for a negative correlation; ^*∗*^*P* < 0.05, ^*∗∗*^*P* < 0.01, and ^*∗∗∗*^*P* < 0.001. (b) The correlation of UBQLN4 expression with activation/inhibition pathways in pan-cancer based on GSCA. UBQLN4: ubiquilin 4; GSCA: Gene Set Cancer Analysis; LAML: acute myeloid leukemia; ALL: acute lymphoblastic leukemia; CML: chronic myelogenous leukemia; GBM: glioblastoma multiforme; LUAD: lung adenocarcinoma; MEL: melanoma; RCC: renal cell carcinoma; BRCA: breast invasive carcinoma; PC: prostate cancer; HNSCC: head and neck cancer; OV: ovarian carcinoma; CRC: colorectal cancer.

CancerSEA database was performed to identify the functional state of UBQLN4 across various cancers at the single-cell level. As shown in Figure 8(a), UBQLN4 was positively related to angiogenesis and negatively correlated with cell cycle, EMT, and quiescence in PC. UBQLN4 was negatively correlated with angiogenesis, DNA damage, and proliferation in OV. However, no significant functional state correlated with UBQLN4 was found in RCC.

Moreover, the role of UBQLN4 in cancer-related pathways in pan-cancer was analyzed using GSCA database. The global percentage in Figure 8(b) indicated that UBQLN4 had a complete inhibitory effect of PI3K/AKT, RAS/MAPK, RTK, TSC/mTOR, and a complete activation of apoptosis.

### 3.8. Correlation and Functional Analyses between UBQLN4 and Other Members in UBQLNs Family

To explore the correlation between UBQLN4 and other members in UBQLNs family (UBQLN1, UBQLN2, UBQLN3, and UBQLNL), coexpression analysis was performed. The heatmap indicated that UBQLN4 was positively related to UBQLN1 and UBQLN2 in most cancers with statistical significance (Supplementary Figure 6(a)), while few correlations were found between UBQLN4 and the remaining two members (UBQLN3 and UBQLNL). Moreover, the role of UBQLNs family in cancer-related pathways in pan-cancer was analyzed using GSCA database to differentiate the function between UBQLN4 and other members in UBQLNs family. The global percentage in Supplementary Figure 6(b) illustrates the functional comparison pattern among UBQLNs family. Similar effects of apoptosis, cell cycle, EMT, hormone ER, PI3K/AKT, RAS/MAPK, and TSC/mTOR were found between UBQLN1 and UBQLN4 in pan-cancer.

### 3.9. Association between UBQLN4 mRNA Expression and Immunotherapy and Immune Microenvironment in Pan-Cancer Based on TCGA Database

**Figure 9:**
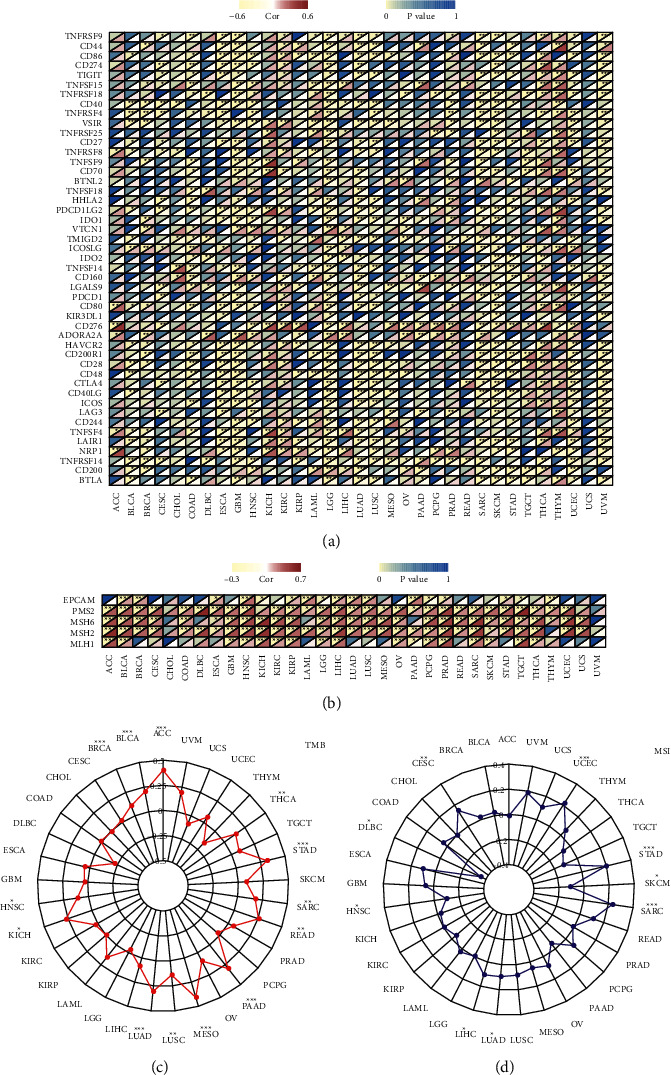
The relationship between UBQLN4 and immune checkpoints, MMR, TMB, and MSI based on TCGA database. Heatmap indicated the correlation between UBQLN4 and known immune checkpoints (a) and known MMR-related genes (b). Correlation between UBQLN4 and TMB (c) and MSI (d). ^*∗*^*P* < 0.05, ^*∗∗*^*P* < 0.01, and ^*∗∗∗*^*P* < 0.001. UBQLN4: ubiquilin 4; MMR: mismatch repair; TMB: tumor mutational burden; MSI: microsatellite instability; TCGA : The Cancer Genome Atlas; ACC: adrenocortical carcinoma; BLCA: bladder urothelial carcinoma; BRCA: breast invasive carcinoma; CESC: cervical squamous cell carcinoma and endocervical adenocarcinoma; CHOL: cholangiocarcinoma; COAD: colon adenocarcinoma; DLBC: lymphoid neoplasm diffuse large B-cell lymphoma; ESCA: esophageal carcinoma; GBM: glioblastoma multiforme; HNSC: head and neck squamous cell carcinoma; KICH: kidney chromophobe; KIRC: kidney renal clear cell carcinoma; KIRP: kidney renal papillary cell carcinoma; LAML: acute myeloid leukemia; LGG: brain lower grade glioma; LIHC: liver hepatocellular carcinoma; LUAD: lung adenocarcinoma; LUSC: lung squamous cell carcinoma; MESO: mesothelioma; OV: ovarian serous cystadenocarcinoma; PAAD: pancreatic adenocarcinoma; PCPG: pheochromocytoma and paraganglioma; PRAD: prostate adenocarcinoma; READ: rectum adenocarcinoma; SARC: sarcoma; SKCM: skin cutaneous melanoma; STAD, stomach adenocarcinoma; TGCT: testicular germ cell tumor; THCA: thyroid carcinoma; THYM: thymoma; UCEC: uterine corpus endometrial carcinoma; UCS: uterine carcinosarcoma; UVM: uveal melanoma.

The coexpression analyses of UBQLN4 mRNA and immune checkpoints and MMR signatures in pan-cancer were performed based on TCGA database. As shown in Figure 9(a), the statistical correlation significance between UBQLN4 mRNA and immune checkpoints existed in COAD, GBM, LGG, LUAD, LUSC, SARC, SKCM, UCEC, and UVM. Moreover, UBQLN4 mRNA were significantly positively correlated with MMR signatures (EPCAM, PMS2, MSH6, MSH2, and MLH1) in the majority of cancer types (Figure 9(b)). Furthermore, the correlation between UBQLN4 and TMB was also found in ACC, BLCA, BRCA, HNSC, KICH, LUAD, LUSC, MESO, pancreatic adenocarcinoma (PAAD), READ, SARC, STAD, and THCA (Figure 9(c)). And the correlation between UBQLN4 and MSI was also found in CESC, DLBC, HNSC, LIHC, LUAD, SARC, SKCM, STAD, and UCEC (Figure 9(d)).

**Figure 10:**
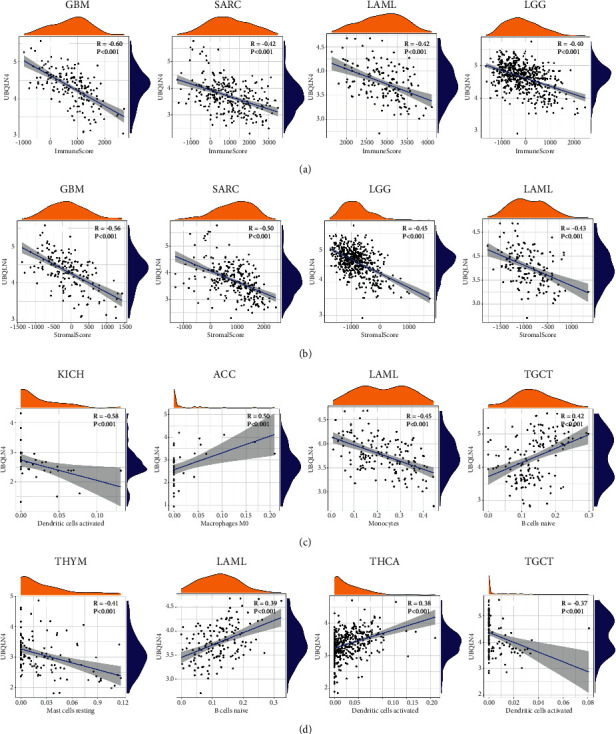
The relationship between UBQLN4 and immune microenvironment. The correlation between UBQLN4 and immune scores (a), stromal scores (b), and immune cells (c) in different cancers with statistical significance. UBQLN4: ubiquilin 4; GBM: glioblastoma multiforme; SARC: sarcoma; LAML: acute myeloid leukemia; LGG: brain lower grade glioma; KICH: kidney chromophobe; ACC: adrenocortical carcinoma; TGCT: testicular germ cell tumor; THYM: thymoma; THCA: thyroid carcinoma.

The correlation between UBQLN4 mRNA and immune microenvironment was also analyzed, and four cancers (GBM, SARC, LAML, and LGG) with the highest immune score and stromal score are illustrated in Figures 10(a) and 10(b). Moreover, the immune score and stromal score of UBQLN4 in the rest cancers are shown in Supplementary Table 7. Almost in all tumors, UBQLN4 mRNA was negatively correlated with immune score and stromal score except THCA. Furthermore, we also explored the relationship between UBQLN4 mRNA and immune cells infiltration (Supplementary Table 8), and the top eight correlations are shown in Figure 10(c).

### 3.10. Correlation between UBQLN4 CNV and Methylation and Immune Microenvironment Based on GSCA Database

**Figure 11:**
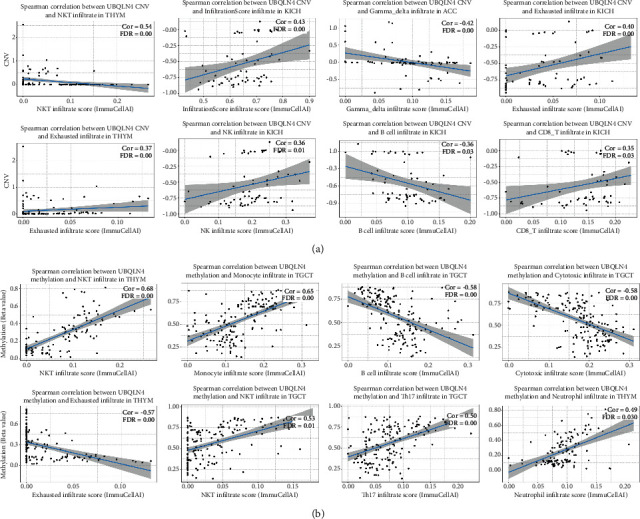
The relationship between immune cells and UBQLN4 CNV and methylation in pan-cancer based on GSCA. The top eight with the highest correlation scores of Spearman correlation between immune cells and UBQLN4 CNV (a) and methylation (b) in different cancers with significance (FDR < 0.05). UBQLN4: ubiquilin 4; CNV: copy number variation; GSCA: Gene Set Cancer Analysis; THYM: thymoma; KICH: kidney chromophobe; ACC: adrenocortical carcinoma; TGCT: testicular germ cell tumor.

Spearman correlation between UBQLN4 CNV and methylation and immune cells infiltrate in pan-cancer was performed using GSCA database. And the top eight with the highest correlation scores are shown in Figures 11(a) and 11(b).

### 3.11. Drug Sensitivity Analysis

The association between the anticancer drugs sensitivity and UBQLN4 mRNA at multiomics level was determined through RNAactDrug database. As shown in Table 1, Quizartinib, 5-Fluorouracil, Masitinib, Zibotentan, Vorinostat, and Alectinib were negatively correlated with UBQLN4 mRNA expression; Dabrafenib, Gemcitabine, Lenalidomide, Veliparib, Vismodegib, Olaparib, Linifanib, and Etoposide were positively correlated with UBQLN4 CNV; Docetaxel and Cetuximab were positively correlated with UBQLN4 mRNA expression.

## 4. Discussion

To our knowledge, this study firstly facilitated multiomics analysis of UBQLN4 in pan-cancer. Firstly, we explored the significance of UBQLN4 in transcriptomics using Oncomine, and TCGA and GTEx were applied for verification. Secondly, we performed proteomics analyses to verify these transcriptomics results based on HPA and CPTAC database. Thirdly, the prognostic value of UBQLN4 was also explored by TCGA database and verified using GEO database. Fourthly, the significance of UBQLN4 in other omics such as mutation, CNV, methylation, and m^6^A methylation was also identified based on multiple databases. Fifthly, we explored the potential functions and pathways of UBQLN4 in pan-cancer based on CancerSEA and GSCA database. Last but not least, the correlation between UBQLN4 and immunotherapy, immune microenvironment, chemotherapy, and targeted therapy at multiomics level was analyzed by multiple databases. Ultimately, we concluded that UBQLN4 was a promising prognostic biomarker of immune-related therapy in pan-cancer. Notably, the reliability of the results would be assured by these effective bioinformatics analyses and repeated verification based on multiomics and multiple databases.

Previous studies have reported that UBQLN4 was overexpressed in neuroblastoma, melanoma, and HCC, related to poor OS [4,8]. Our results were consistent with that UBQLN4 was overexpressed in mostly all cancer types compared with normal tissues, which was also verified at proteomics. Notably, higher expression of UBQLN4 predicted better OS, DSS, and PFI in GBM and LGG, indicating the heterology among various cancers. It was reported that UBQLN4 could be involved in autophagy by regulating UBQLN1 [34], which was also a member of UBQLNs family. Furthermore, increased UBQLN1 expression level contributed to protecting neurons from toxicity caused by protein misfolding [35]. Thus, we speculated that UBQLN4 might be involved in the regulation of neurologic tumors through interacting with UBQLN1. Actually, the mechanism of UBQLN4 in cancers was less explored compared with other members in UBQLNs family. Since a high degree of similarity among UBQLNs family, we performed the correlation and function analyses among them to further differentiate and understand the definitive role of UBQLN4. Our results indicated that UBQLN1 and UBQLN4 played a similar role in some cancer-related pathways, such as apoptosis, cell cycle, EMT, hormone ER, PI3K/AKT, RAS/MAPK, and TSC/mTOR. Interestingly, UBQLN1 was reported to regulate autophagic flux through mTOR signaling [36], which was in line with our results. Thus, UBQLN4 might also be involved in autophagy through mTOR signaling. However, further experimental analysis was also necessary to confirm these speculations.

Recently, the mutation of UBQLN4 was reported to result in genomic instability [4], which was one of the features of tumors. Yielding similar results, our study demonstrated that UBQLN4 mutation was associated with poor prognosis in several tumors. CNV was one of the primary forms of cancer genetic alteration, and CNV patterns in different cancer types varied [37]. As the stability of CNV, it was identified as better biomarker than gene expression characteristics in the field of cancer diagnosis and subtyping [38]. In this study, UBQLN4 wild type revealed better OS and PFS than CNV type in KIRP, UCEC, and LUSC, suggesting the prognostic value of UBQLN4 CNV.

Increasing attention has been paid to the role of epigenetics in recent years, especially methylation, although genetic alterations are essential factors affecting the occurrence and development of tumors [39]. Our results found that UBQLN4 was positively correlated with methyltransferase-related genes in most tumors, and UBQLN4 methylation was associated with UBQLN4 mRNA, indicating the correlation between UBQLN4 methylation and tumor genetics. Interestingly, OS difference between high and low methylation of UBQLN4 was found in KIRP, suggesting the prognostic value of UBQLN4 methylation. Additionally, differences and prognostic significance of UBQLN4 mRNA expression, mutation, and CNV were shown in KIRP, suggesting that UBQLN4 may be a meaningful molecular marker in KIRP. Besides DNA methylation, the RNA internal modification also matters in tumors, especially m^6^A methylation [40]. In this study, we found that UBQLN4 was positively correlated with m^6^A-related genes in most tumors. Furthermore, Human_m6A_14897 was found comethylated with HIF1A, suggesting that it may be involved in the hypoxia pathway.

As we know, immune checkpoint inhibitors are great innovations in tumor therapy in the past decade, in which PD-L1, PD-1, and CTLA4 are the most widely studied checkpoints [41]. However, not all people could benefit from immunotherapy, and the screening of dominant group becomes crucial [42]. In previous clinical practice, PD-L1 overexpression, deficient MMR, MSI-high, and TMB-high indicated a better effect of immunotherapy [43]. Our results showed that there was a significant correlation between UBQLN4 and PD-L1, TMB, MSI, MMR in a variety of tumors, suggesting that UBQLN4 may be used as a new indicator for screening the dominant population in immunotherapy. Among 13 tumor types, high expression of UBQLN4 was associated with high TMB, predicting a better response to immunotherapy. In addition, positive correlations between UBQLN4 and MSI in UCEC, STAD, SARC, LUAD, LIHC, and CESC and negative correlations in SKCM, HNSC, and DLBC were also observed. Moreover, UBQLN4 was positively correlated with PD-L1 in most tumors, suggesting its important role in predicting the efficacy of immunotherapy. Nevertheless, tumor microenvironment, including immune cells and stromal cells, is also closely related to immunotherapy [44,45]. Our study found that higher expression of UBQLN4 was significantly correlated with lower immune and stromal scores in most tumors, suggesting that UBQLN4 may participate in tumor progression by regulating the immune microenvironment. To further explore the relationship between UBQLN4 and tumor microenvironment, we estimated the correlation between UBQLN4 expression, CNV, methylation, and various immune cells in different tumors. Unfortunately, there is still a lack of research on the specific mechanism between UBQLN4 and immune cells. However, the role of immune cells in the progression and treatment of various tumors has been reported. Activated dendritic cells were applied in tumor immunotherapy as for its function on eliminating tumors by improving the body's immune response [46]. Our results indicated that activated dendritic cells were significantly correlated with the expression of UBQLN4 in a variety of tumors, supporting the role of UBQLN4 in immunotherapy. Therefore, it is necessary to further study the relationship between UBQLN4 and immune cells, and UBQLN4 may become a new potential biomarker for screening the dominant population of immunotherapy.

In addition to immunotherapy, UBQLN4 also showed the potential of screening dominant groups in radiotherapy, chemotherapy, and targeted therapy. For further exploration of the relationship between UBQLN4 and oncotherapy, the drug sensitivity analysis of UBQLN4 in various anticancer drugs was performed based on the multiomics data. It was reported that overexpression of UBQLN4 in neuroblastoma cells increased the sensitivity of PARP inhibitors [4]. However, Murakami et al. found that knocking down UBQLN4 in esophageal squamous cell carcinoma increased cisplatin sensitivity [7], suggesting different roles of UBQLN4 played in various drugs. In line with previous studies, our results showed that there was a positive correlation between UBQLN4 CNV and olaparib sensitivity. Furthermore, UBQLN4 expression and CNV were significantly correlated with the sensitivity of various chemotherapeutic drugs and targeted drugs, demonstrating the potential of UBQLN4 in response evaluation for oncotherapy. Additionally, Jachimowicz et al. reported that the deletion of UBQLN4 significantly increased the sensitivity of U-2 OS cells to radioactive analog drug [4], which revealed that UBQLN4 might be a promising biomarker for predicting radiosensitivity. However, the research on UBQLN4 in tumor treatment is still limited, and further research is urgent.

Finally, we analyzed the mechanism of UBQLN4 in pan-cancer. Previous studies mainly focused on the role of UBQLN4 in maintaining protein homeostasis, which was one of the mechanisms of tumorigenesis [47]. Recently, the relationship between UBQLN4 and genomic instability has become a hotspot [48]. It is well acknowledged that the accumulation of DNA damage is an essential mechanism affecting genomic instability, and DNA double-strand break (DSB) is the most serious type of DNA damage [49]. Furthermore, accurate homologous recombination and error-prone nonhomologous end joining (NHEJ) pathways are the main approaches of DSB repair [50]. It was reported that serine at site 318 of UBQLN4 (pS318/UBQLN4) would be phosphorylated under the catalysis of ATM when DNA was damaged [4]. Phosphorylated UBQLN4 was recruited to the DNA damage site and promotes proteasome degradation through the specific interaction between its UBA domain and ubiquitinated MRE11, which was a homologous recombination-mediated DSB repair (HRR-) related factor [51]. Therefore, when UBQLN4 was highly expressed, the DSB repair pathway tilted to NHEJ, which promoted genomic instability. Similar results were obtained in our study, UBQLN4 mainly had an activating role in DNA damage response and participated in DNA repair in some tumors. Since DNA damage is an important mechanism of radiotherapy and chemotherapy [52], it may be one of the mechanisms of UBQLN4 affecting tumor therapy. Furthermore, the mechanism of UBQLN4 in some tumors has also been described in previous studies. It was reported that the overexpression of UBQLN4 in gastric cancer cells could induce cell cycle arrest and activate the p53/p21 axis, thus significantly inhibiting the proliferation of gastric cancer cells [9]. In hepatocellular carcinoma, UBQLN4 promoted tumor proliferation and invasion by regulating wnt- *β*-catenin pathway, while miR-370 could downregulate UBQLN4 to inhibit tumor progression [8]. Similarly, our study also found that UBQLN4 was involved in cell cycle, proliferation, and apoptosis. However, there is still a lack of further experiments in more tumors to understand the specific mechanism of UBQLN4.

## 5. Conclusions

In conclusion, our study estimated the role of UBQLN4 in pan-cancer for the first time. Based on the comprehensive analysis of multiomics data, we identified UBQLN4 as a potential molecular biomarker for prognosis and treatment in oncotherapy. However, since our research was based on bioinformatics analysis, further clinical and experimental analyses are emergent to verify our results.

## Figures and Tables

**Table 1:** The detailed information between anticancer drug sensitivity and UBQLN4 mRNA based on multiomics data.

Drug	RNA type	RNA molecule	Omics	Source	Pearson.Cor	Pearson.FDR	Spearman.Cor	Spearman.FDR
Dabrafenib	mRNA	UBQLN4	CNV	GDSC	0.11	*∗∗*	0.16	*∗∗∗*
Quizartinib	mRNA	UBQLN4	Expression	GDSC	−0.12	*∗∗∗*	−0.14	*∗∗∗*
5-Fluorouracil	mRNA	UBQLN4	Expression	GDSC	−0.12	*∗∗∗*	−0.13	*∗∗∗*
Gemcitabine	mRNA	UBQLN4	CNV	GDSC	0.11	*∗∗*	0.12	*∗∗*
Lenalidomide	mRNA	UBQLN4	CNV	GDSC	0.12	*∗∗*	0.12	*∗∗*
Veliparib	mRNA	UBQLN4	CNV	GDSC	0.12	*∗∗*	0.12	*∗∗*
Vismodegib	mRNA	UBQLN4	CNV	GDSC	0.12	*∗∗*	0.12	*∗∗∗*
Docetaxel	mRNA	UBQLN4	Expression	GDSC	0.12	*∗∗∗*	0.11	*∗∗*
Cetuximab	mRNA	UBQLN4	Expression	GDSC	0.11	*∗∗*	0.1	*∗*
Olaparib	mRNA	UBQLN4	CNV	GDSC	0.1	*∗∗*	0.1	*∗∗*
Linifanib	mRNA	UBQLN4	CNV	GDSC	0.1	*∗∗*	NA	NA
Etoposide	mRNA	UBQLN4	CNV	GDSC	NA	NA	0.08	*∗*
Masitinib	mRNA	UBQLN4	Expression	GDSC	NA	NA	−0.07	*∗*
Zibotentan	mRNA	UBQLN4	Expression	GDSC	NA	NA	−0.07	*∗*
Vorinostat	mRNA	UBQLN4	Expression	GDSC	NA	NA	−0.08	*∗*
Alectinib	mRNA	UBQLN4	Expression	GDSC	NA	NA	−0.09	*∗∗*

UBQLN4: ubiquilin 4; CNV: copy number variation; GDSC : Genomics of Drug Sensitivity in Cancer; NA: not available; ^*∗*^*P* < 0.05, ^*∗∗*^*P* < 0.01, and ^*∗∗∗*^*P* < 0.001.

## Data Availability

The data used to support the findings of this study are available in public databases that were included within this article.

## Supplementary Materials

Supplementary Materials *Supplementary Figure 1.* The DSS analysis of UBQLN4 mRNA in pan-cancer based on TCGA database. The Kaplan–Meier curves of UBQLN4 in ACC (A), GBM (B), KICH (C), LGG (D), LIHC (E), MESO (F), PCPG (G), SARC (H), SKCM (I), and UVM (J) with significance based on TCGA database. (K) The cox regression analysis for DSS and UBQLN4 expression in 33 cancer types based on TCGA database. Abbreviations: DSS: disease-specific survival; UBQLN4: ubiquilin 4; TCGA: The Cancer Genome Atlas; ACC: adrenocortical carcinoma; BLCA: bladder urothelial carcinoma; BRCA: breast invasive carcinoma; CESC: cervical squamous cell carcinoma and endocervical adenocarcinoma; CHOL: cholangiocarcinoma; COAD: colon adenocarcinoma; DLBC: lymphoid neoplasm diffuse large B-cell lymphoma; ESCA: esophageal carcinoma; GBM: glioblastoma multiforme; HNSC: head and neck squamous cell carcinoma; KICH: kidney chromophobe; KIRC: kidney renal clear cell carcinoma; KIRP: kidney renal papillary cell carcinoma; LAML: acute myeloid leukemia; LGG: brain lower grade glioma; LIHC: liver hepatocellular carcinoma; LUAD: lung adenocarcinoma; LUSC: lung squamous cell carcinoma; MESO: mesothelioma; OV: ovarian serous cystadenocarcinoma; PAAD: pancreatic adenocarcinoma; PCPG: pheochromocytoma and paraganglioma; PRAD: prostate adenocarcinoma; READ: rectum adenocarcinoma; SARC: sarcoma; SKCM: skin cutaneous melanoma; STAD, stomach adenocarcinoma; TGCT: testicular germ cell tumor; THCA: thyroid carcinoma; THYM: thymoma; UCEC: uterine corpus endometrial carcinoma; UCS: uterine carcinosarcoma; UVM: uveal melanoma. *Supplementary Figure 2*. The DFI analysis of UBQLN4 mRNA in pan-cancer based on TCGA database. The Kaplan–Meier curves of UBQLN4 in READ (A), SARC (B), and UCEC (C) with significance based on TCGA database. (D) The cox regression analysis for DFI and UBQLN4 expression in 33 cancer types based on TCGA database. Abbreviations: DFI: disease-free interval; UBQLN4: ubiquilin 4; TCGA: The Cancer Genome Atlas; ACC: adrenocortical carcinoma; BLCA: bladder urothelial carcinoma; BRCA: breast invasive carcinoma; CESC: cervical squamous cell carcinoma and endocervical adenocarcinoma; CHOL: cholangiocarcinoma; COAD: colon adenocarcinoma; DLBC: lymphoid neoplasm diffuse large B-cell lymphoma; ESCA: esophageal carcinoma; GBM: glioblastoma multiforme; HNSC: head and neck squamous cell carcinoma; KICH: kidney chromophobe; KIRC: kidney renal clear cell carcinoma; KIRP: kidney renal papillary cell carcinoma; LAML: acute myeloid leukemia; LGG: brain lower grade glioma; LIHC: liver hepatocellular carcinoma; LUAD: lung adenocarcinoma; LUSC: lung squamous cell carcinoma; MESO: mesothelioma; OV: ovarian serous cystadenocarcinoma; PAAD: pancreatic adenocarcinoma; PCPG: pheochromocytoma and paraganglioma; PRAD: prostate adenocarcinoma; READ: rectum adenocarcinoma; SARC: sarcoma; SKCM: skin cutaneous melanoma; STAD, stomach adenocarcinoma; TGCT: testicular germ cell tumor; THCA: thyroid carcinoma; THYM: thymoma; UCEC: uterine corpus endometrial carcinoma; UCS: uterine carcinosarcoma; UVM: uveal melanoma. *Supplementary Figure 3*. The PFI analysis of UBQLN4 mRNA in pan-cancer based on TCGA database. The Kaplan–Meier curves of UBQLN4 in ACC (A), GBM (B), LGG (C), LIHC (D), MESO (E), SARC (F), THYM (G), and UCEC (H) with significance based on TCGA database. (I) The cox regression analysis for PFI and UBQLN4 expression in 33 cancer types based on TCGA database. Abbreviations: PFI: progression-free interval; UBQLN4: ubiquilin 4; TCGA: The Cancer Genome Atlas; ACC: adrenocortical carcinoma; BLCA: bladder urothelial carcinoma; BRCA: breast invasive carcinoma; CESC: cervical squamous cell carcinoma and endocervical adenocarcinoma; CHOL: cholangiocarcinoma; COAD: colon adenocarcinoma; DLBC: lymphoid neoplasm diffuse large B-cell lymphoma; ESCA: esophageal carcinoma; GBM: glioblastoma multiforme; HNSC: head and neck squamous cell carcinoma; KICH: kidney chromophobe; KIRC: kidney renal clear cell carcinoma; KIRP: kidney renal papillary cell carcinoma; LAML: acute myeloid leukemia; LGG: brain lower grade glioma; LIHC: liver hepatocellular carcinoma; LUAD: lung adenocarcinoma; LUSC: lung squamous cell carcinoma; MESO: mesothelioma; OV: ovarian serous cystadenocarcinoma; PAAD: pancreatic adenocarcinoma; PCPG: pheochromocytoma and paraganglioma; PRAD: prostate adenocarcinoma; READ: rectum adenocarcinoma; SARC: sarcoma; SKCM: skin cutaneous melanoma; STAD, stomach adenocarcinoma; TGCT: testicular germ cell tumor; THCA: thyroid carcinoma; THYM: thymoma; UCEC: uterine corpus endometrial carcinoma; UCS: uterine carcinosarcoma; UVM: uveal melanoma. *Supplementary Figure 4*. The survival analysis of UBQLN4 CNV based on CNV module in GSCA. The OS curves of UBQLN4 CNV in ACC (A), KIRP (B), LIHC (C), LUSC (D), and UCEC (E), and the PFS curves of UBQLN4 CNV in ACC (F), KIRP (G), LUSC (H), THCA (I), and UCEC (J). Abbreviations: UBQLN4: ubiquilin 4; CNV: copy number variation; GSCA: Gene Set Cancer Analysis; OS: overall survival; PFS: progression-free survival; ACC: adrenocortical carcinoma; KIRP: kidney renal papillary cell carcinoma; LIHC: liver hepatocellular carcinoma; LUSC: lung squamous cell carcinoma; UCEC: uterine corpus endometrial carcinoma; THCA: thyroid carcinoma. *Supplementary Figure 5*. The survival analysis of UBQLN4 methylation based on Methylation module in GSCA. UBQLN4 hypermethylation in KIRP(A) and UBQLN4 hypomethylation in UVM (B) indicated better OS. Abbreviations: UBQLN4: ubiquilin 4; GSCA: Gene Set Cancer Analysis; KIRP: kidney renal papillary cell carcinoma; UVM: uveal melanoma; OS: overall survival. *Supplementary Figure 6*. The correlation and functional analyses between UBQLN4 and other members in UBQLNs family. (A) Correlation between UBQLN4 and other members in UBQLNs family (UBQLN1, UBQLN2, UBQLN3, and UBQLNL). (B) The correlation of UBQLNs family (UBQLN1, UBQLN2, UBQLN3, UBQLN4, and UBQLNL) expression with activation/inhibition pathways in pan-cancer based on GSCA. Abbreviations: UBQLN4: ubiquilin 4; GSCA: Gene Set Cancer Analysis; ACC: adrenocortical carcinoma; BLCA: bladder urothelial carcinoma; BRCA: breast invasive carcinoma; CESC: cervical squamous cell carcinoma and endocervical adenocarcinoma; CHOL: cholangiocarcinoma; COAD: colon adenocarcinoma; DLBC: lymphoid neoplasm diffuse large B-cell lymphoma; ESCA: esophageal carcinoma; GBM: glioblastoma multiforme; HNSC: head and neck squamous cell carcinoma; KICH: kidney chromophobe; KIRC: kidney renal clear cell carcinoma; KIRP: kidney renal papillary cell carcinoma; LAML: acute myeloid leukemia; LGG: brain lower grade glioma; LIHC: liver hepatocellular carcinoma; LUAD: lung adenocarcinoma; LUSC: lung squamous cell carcinoma; MESO: mesothelioma; OV: ovarian serous cystadenocarcinoma; PAAD: pancreatic adenocarcinoma; PCPG: pheochromocytoma and paraganglioma; PRAD: prostate adenocarcinoma; READ: rectum adenocarcinoma; SARC: sarcoma; SKCM: skin cutaneous melanoma; STAD, stomach adenocarcinoma; TGCT: testicular germ cell tumor; THCA: thyroid carcinoma; THYM: thymoma; UCEC: uterine corpus endometrial carcinoma; UCS: uterine carcinosarcoma; UVM: uveal melanoma; NA: not available. *Supplementary Table 1*. The detailed information of 33 cancer types. *Supplementary Table 2*. The detailed ratio of UBQLN4 CNV types in every cancer type. *Supplementary Table 3*. Correlation between UBQLN4 CNV and mRNA expression in every cancer type. *Supplementary Table 4*. Correlation between UBQLN4 methylation and mRNA expression in every cancer type. *Supplementary Table 5*. Detailed information of m^6^A records related to UBQLN4. *Supplementary Table 6*. Detailed information of human_m6A_14897, including the RNA Binding Region, miRNA-Targets, predicted GO functions, and comethylated m^6^A sites. *Supplementary Table 7*. The immune score and stromal score of UBQLN4 in the rest of cancers with statistical significance. *Supplementary Table 8*. The relationship between UBQLN4 mRNA and immune cells infiltration.
