# Supplementary material for: A Comprehensive Multiomics Analysis Identified Ubiquilin 4 as a Promising Prognostic Biomarker of Immune-Related Therapy in Pan-Cancer
Source: J Oncol. 2021 Sep 7;2021:7404927. doi: 10.1155/2021/7404927 (PMC8443395; doi:10.1155/2021/7404927)
Supplement: Supplementary Materials — Supplementary Figure 1. The DSS analysis of UBQLN4 mRNA in pan-cancer based on TCGA database. The Kaplan–Meier curves of UBQLN4 in ACC (A), GBM (B), KICH (C), LGG (D), LIHC (E), MESO (F), PCPG (G), SARC (H), SKCM (I), and UVM (J) with significance based on TCGA database. (K) The cox regression analysis for DSS and UBQLN4 expression in 33 cancer types based on TCGA database. Abbreviations: DSS: disease-specific survival; UBQLN4: ubiquilin 4; TCGA: The Cancer Genome Atlas; ACC: adrenocortical carcinoma; BLCA: bladder urothelial carcinoma; BRCA: breast invasive carcinoma; CESC: cervical squamous cell carcinoma and endocervical adenocarcinoma; CHOL: cholangiocarcinoma; COAD: colon adenocarcinoma; DLBC: lymphoid neoplasm diffuse large B-cell lymphoma; ESCA: esophageal carcinoma; GBM: glioblastoma multiforme; HNSC: head and neck squamous cell carcinoma; KICH: kidney chromophobe; KIRC: kidney renal clear cell carcinoma; KIRP: kidney renal papillary cell carcinoma; LAML: acute myeloid leukemia; LGG: brain lower grade glioma; LIHC: liver hepatocellular carcinoma; LUAD: lung adenocarcinoma; LUSC: lung squamous cell carcinoma; MESO: mesothelioma; OV: ovarian serous cystadenocarcinoma; PAAD: pancreatic adenocarcinoma; PCPG: pheochromocytoma and paraganglioma; PRAD: prostate adenocarcinoma; READ: rectum adenocarcinoma; SARC: sarcoma; SKCM: skin cutaneous melanoma; STAD, stomach adenocarcinoma; TGCT: testicular germ cell tumor; THCA: thyroid carcinoma; THYM: thymoma; UCEC: uterine corpus endometrial carcinoma; UCS: uterine carcinosarcoma; UVM: uveal melanoma. Supplementary Figure 2. The DFI analysis of UBQLN4 mRNA in pan-cancer based on TCGA database. The Kaplan–Meier curves of UBQLN4 in READ (A), SARC (B), and UCEC (C) with significance based on TCGA database. (D) The cox regression analysis for DFI and UBQLN4 expression in 33 cancer types based on TCGA database. Abbreviations: DFI: disease-free interval; UBQLN4: ubiquilin 4; TCGA: The Cancer Genome Atlas; ACC: adrenocortical ca [file 7404927.f1.zip › 7404927.f1/Supplementary Table 2.pdf]

Supplementary Table 2. The detailed ratio of UBQLN4 CNV types in every cancer type.

Cancer type	Total amp.(%)	Total dele.(%)	Hete. amp.(%)	Hete. dele.(%)	Homo. amp.(%)	Homo. dele.(%)
ACC	16.67	14.44	13.33	14.44	3.33	0
BLCA	45.1	6.37	38.73	6.13	6.37	0.25
BRCA	72.96	1.76	62.13	1.76	10.83	0
CESC	53.56	2.37	50.51	2.37	3.05	0
CHOL	63.89	0	50	0	13.89	0
COAD	20.18	9.53	19.73	9.53	0.44	0
DLBC	25	4.17	20.83	4.17	4.17	0
ESCA	48.37	4.35	45.11	4.35	3.26	0
GBM	18.54	1.56	18.02	1.56	0.52	0
HNSC	24.9	7.28	23.56	7.28	1.34	0
KICH	1.52	77.27	1.52	77.27	0	0
KIRC	12.12	6.44	11.55	6.44	0.57	0
KIRP	5.56	6.6	5.56	6.6	0	0
LAML	1.05	0	1.05	0	0	0
LGG	7.41	1.95	6.43	1.95	0.97	0
LIHC	74.86	0.27	61.62	0.27	13.24	0
LUAD	73.06	1.74	61.43	1.74	11.63	0
LUSC	52.3	4.59	48.1	4.59	4.19	0
MESO	29.89	1.15	27.59	1.15	2.3	0
OV	61.66	2.25	51.47	2.25	10.19	0
PAAD	33.15	3.26	29.89	3.26	3.26	0
PCPG	16.67	8.64	13.58	8.64	3.09	0
PRAD	6.71	3.25	5.89	2.03	0.81	1.22
READ	28.48	7.88	27.88	7.88	0.61	0
SARC	36.58	7.39	31.91	7.39	4.67	0
SKCM	54.22	3.54	51.5	3.54	2.72	0
STAD	36.05	3.63	33.79	2.95	2.27	0.68
TGCT	45.33	0.67	45.33	0.67	0	0
THCA	5.41	0.6	5.41	0.6	0	0
THYM	19.51	0	18.7	0	0.81	0
UCEC	47.68	0.19	38.78	0.19	8.91	0
UCS	73.21	5.36	53.57	5.36	19.64	0
UVM	10	0	10	0	0	0

Abbrevations: UBQLN4: ubiquilin 4; CNV: copy number variation; amp: amplification; hete: heterozygous; Hete amp: Heterozygous amplification; Hete del: Heterozygous deletion; Homo amp: Homozygous amplification; Homo del: Homozygous deletion; ACC: adrenocortical carcinoma; BLCA: bladder urothelial carcinoma; BRCA: breast invasive carcinoma; CESC: cervical squamous cell carcinoma and endocervical adenocarcinoma; CHOL:

cholangiocarcinoma; COAD: colon adenocarcinoma; DLBC: lymphoid neoplasm diffuse large B-cell lymphoma; ESCA: esophageal carcinoma; GBM: glioblastoma multiforme; HNSC: head and neck squamous cell carcinoma; KICH: kidney chromophobe; KIRC: kidney renal clear cell carcinoma; KIRP: kidney renal papillary cell carcinoma; LAML: acute myeloid leukemia; LGG: brain lower grade glioma; LIHC: liver hepatocellular carcinoma; LUAD: lung adenocarcinoma; LUSC: lung squamous cell carcinoma; MESO: mesothelioma; OV: ovarian serous cystadenocarcinoma; PAAD: pancreatic adenocarcinoma; PCPG: pheochromocytoma and paraganglioma; PRAD: prostate adenocarcinoma; READ: rectum adenocarcinoma; SARC: sarcoma; SKCM: skin cutaneous melanoma; STAD, stomach adenocarcinoma; TGCT: testicular germ cell tumor; THCA: thyroid carcinoma; THYM: thymoma; UCEC: uterine corpus endometrial carcinoma; UCS: uterine carcinosarcoma; UVM: uveal melanoma.
