# Supplementary material for: A Comprehensive Multiomics Analysis Identified Ubiquilin 4 as a Promising Prognostic Biomarker of Immune-Related Therapy in Pan-Cancer
Source: J Oncol. 2021 Sep 7;2021:7404927. doi: 10.1155/2021/7404927 (PMC8443395; doi:10.1155/2021/7404927)
Supplement: Supplementary Materials — Supplementary Figure 1. The DSS analysis of UBQLN4 mRNA in pan-cancer based on TCGA database. The Kaplan–Meier curves of UBQLN4 in ACC (A), GBM (B), KICH (C), LGG (D), LIHC (E), MESO (F), PCPG (G), SARC (H), SKCM (I), and UVM (J) with significance based on TCGA database. (K) The cox regression analysis for DSS and UBQLN4 expression in 33 cancer types based on TCGA database. Abbreviations: DSS: disease-specific survival; UBQLN4: ubiquilin 4; TCGA: The Cancer Genome Atlas; ACC: adrenocortical carcinoma; BLCA: bladder urothelial carcinoma; BRCA: breast invasive carcinoma; CESC: cervical squamous cell carcinoma and endocervical adenocarcinoma; CHOL: cholangiocarcinoma; COAD: colon adenocarcinoma; DLBC: lymphoid neoplasm diffuse large B-cell lymphoma; ESCA: esophageal carcinoma; GBM: glioblastoma multiforme; HNSC: head and neck squamous cell carcinoma; KICH: kidney chromophobe; KIRC: kidney renal clear cell carcinoma; KIRP: kidney renal papillary cell carcinoma; LAML: acute myeloid leukemia; LGG: brain lower grade glioma; LIHC: liver hepatocellular carcinoma; LUAD: lung adenocarcinoma; LUSC: lung squamous cell carcinoma; MESO: mesothelioma; OV: ovarian serous cystadenocarcinoma; PAAD: pancreatic adenocarcinoma; PCPG: pheochromocytoma and paraganglioma; PRAD: prostate adenocarcinoma; READ: rectum adenocarcinoma; SARC: sarcoma; SKCM: skin cutaneous melanoma; STAD, stomach adenocarcinoma; TGCT: testicular germ cell tumor; THCA: thyroid carcinoma; THYM: thymoma; UCEC: uterine corpus endometrial carcinoma; UCS: uterine carcinosarcoma; UVM: uveal melanoma. Supplementary Figure 2. The DFI analysis of UBQLN4 mRNA in pan-cancer based on TCGA database. The Kaplan–Meier curves of UBQLN4 in READ (A), SARC (B), and UCEC (C) with significance based on TCGA database. (D) The cox regression analysis for DFI and UBQLN4 expression in 33 cancer types based on TCGA database. Abbreviations: DFI: disease-free interval; UBQLN4: ubiquilin 4; TCGA: The Cancer Genome Atlas; ACC: adrenocortical ca [file 7404927.f1.zip › 7404927.f1/Supplementary Table 3.pdf]

Supplementary Table 3. Correlation between UBQLN4 CNV and mRNA expression in every cancer type.

Cancer type	Spearman correlation	FDR
CHOL	0.81	0
UCS	0.79	0
THYM	0.7	0
LUAD	0.7	0
OV	0.68	0
BRCA	0.66	0
MESO	0.66	0
PAAD	0.65	0
UCEC	0.65	0
LIHC	0.65	0
SKCM	0.65	0
READ	0.63	0
ACC	0.61	0
CESC	0.61	0
SARC	0.6	0
ESCA	0.59	0
BLCA	0.58	0
DLBC	0.57	0
COAD	0.57	0
LUSC	0.56	0
STAD	0.53	0
HNSC	0.51	0
KICH	0.5	0
KIRC	0.37	0
PCPG	0.34	0
THCA	0.34	0
KIRP	0.29	0
LGG	0.24	0
PRAD	0.17	0
UVM	0.13	0.46
TGCT	0.12	0.2
GBM	0.1	0.32
LAML	0.04	0.86

Abbrevations: UBQLN4: ubiquilin 4; CNV: copy number variation; ACC: adrenocortical carcinoma; BLCA: bladder urothelial carcinoma; BRCA: breast invasive carcinoma; CESC: cervical squamous cell carcinoma and endocervical adenocarcinoma; CHOL: cholangiocarcinoma; COAD: colon adenocarcinoma; DLBC: lymphoid neoplasm diffuse large B-cell lymphoma; ESCA: esophageal carcinoma; GBM: glioblastoma multiforme; HNSC: head and neck squamous cell carcinoma; KICH: kidney chromophobe; KIRC: kidney

renal clear cell carcinoma; KIRP: kidney renal papillary cell carcinoma; LAML: acute myeloid leukemia; LGG: brain lower grade glioma; LIHC: liver hepatocellular carcinoma; LUAD: lung adenocarcinoma; LUSC: lung squamous cell carcinoma; MESO: mesothelioma; OV: ovarian serous cystadenocarcinoma; PAAD: pancreatic adenocarcinoma; PCPG: pheochromocytoma and paraganglioma; PRAD: prostate adenocarcinoma; READ: rectum adenocarcinoma; SARC: sarcoma; SKCM: skin cutaneous melanoma; STAD, stomach adenocarcinoma; TGCT: testicular germ cell tumor; THCA: thyroid carcinoma; THYM: thymoma; UCEC: uterine corpus endometrial carcinoma; UCS: uterine carcinosarcoma; UVM: uveal melanoma.
