# Supplementary material for: A Comprehensive Multiomics Analysis Identified Ubiquilin 4 as a Promising Prognostic Biomarker of Immune-Related Therapy in Pan-Cancer
Source: J Oncol. 2021 Sep 7;2021:7404927. doi: 10.1155/2021/7404927 (PMC8443395; doi:10.1155/2021/7404927)
Supplement: Supplementary Materials — Supplementary Figure 1. The DSS analysis of UBQLN4 mRNA in pan-cancer based on TCGA database. The Kaplan–Meier curves of UBQLN4 in ACC (A), GBM (B), KICH (C), LGG (D), LIHC (E), MESO (F), PCPG (G), SARC (H), SKCM (I), and UVM (J) with significance based on TCGA database. (K) The cox regression analysis for DSS and UBQLN4 expression in 33 cancer types based on TCGA database. Abbreviations: DSS: disease-specific survival; UBQLN4: ubiquilin 4; TCGA: The Cancer Genome Atlas; ACC: adrenocortical carcinoma; BLCA: bladder urothelial carcinoma; BRCA: breast invasive carcinoma; CESC: cervical squamous cell carcinoma and endocervical adenocarcinoma; CHOL: cholangiocarcinoma; COAD: colon adenocarcinoma; DLBC: lymphoid neoplasm diffuse large B-cell lymphoma; ESCA: esophageal carcinoma; GBM: glioblastoma multiforme; HNSC: head and neck squamous cell carcinoma; KICH: kidney chromophobe; KIRC: kidney renal clear cell carcinoma; KIRP: kidney renal papillary cell carcinoma; LAML: acute myeloid leukemia; LGG: brain lower grade glioma; LIHC: liver hepatocellular carcinoma; LUAD: lung adenocarcinoma; LUSC: lung squamous cell carcinoma; MESO: mesothelioma; OV: ovarian serous cystadenocarcinoma; PAAD: pancreatic adenocarcinoma; PCPG: pheochromocytoma and paraganglioma; PRAD: prostate adenocarcinoma; READ: rectum adenocarcinoma; SARC: sarcoma; SKCM: skin cutaneous melanoma; STAD, stomach adenocarcinoma; TGCT: testicular germ cell tumor; THCA: thyroid carcinoma; THYM: thymoma; UCEC: uterine corpus endometrial carcinoma; UCS: uterine carcinosarcoma; UVM: uveal melanoma. Supplementary Figure 2. The DFI analysis of UBQLN4 mRNA in pan-cancer based on TCGA database. The Kaplan–Meier curves of UBQLN4 in READ (A), SARC (B), and UCEC (C) with significance based on TCGA database. (D) The cox regression analysis for DFI and UBQLN4 expression in 33 cancer types based on TCGA database. Abbreviations: DFI: disease-free interval; UBQLN4: ubiquilin 4; TCGA: The Cancer Genome Atlas; ACC: adrenocortical ca [file 7404927.f1.zip › 7404927.f1/Supplementary Table 5.pdf]

Supplementary Table 5. Detailed information of m6A records related to UBQLN4.

m6A ID	Position	Gene Region	PubMed ID	GSE	Technique	Cell line/Tissue	GO Prediction	RBP	miRNA Target
human_m6A_14888	chr1:156005329	3'UTR	31281898	GSE125240	m6A-REF-seq	HEK293	NA	10	1
human_m6A_14889	chr1:156005620	3'UTR	31257032	GSE122961	MAZTER-seq	HEK293T	NA	NA	NA
human_m6A_14890	chr1:156005809	3'UTR	31279658	GSE122948	miCLIP	HEK293T	521	NA	NA
human_m6A_14891	chr1:156006126	3'UTR	31257032	GSE122961	MAZTER-seq	HEK293T	NA	9	NA
human_m6A_14892	chr1:156006136	3'UTR	31279658	GSE122948	miCLIP	HEK293T	NA	11	NA
human_m6A_14893	chr1:156006148	3'UTR	31281898	GSE125240	m6A-REF-seq	brain	NA	10	NA
human_m6A_14894	chr1:156006272	3'UTR	31281898	GSE125240	m6A-REF-seq	brain	NA	1	NA
human_m6A_14895	chr1:156006411	3'UTR	31257032	GSE122961	MAZTER-seq	ESC	NA	NA	2
human_m6A_14896	chr1:156006505	3'UTR	25491922	GSE54921	PA-m6A-seq	HeLa	NA	NA	9
human_m6A_14897	chr1:156006651	3'UTR	31279658	GSE122948	miCLIP	HEK293T	475	7	2
human_m6A_14898	chr1:156006815	exonic	25491922	GSE54921	PA-m6A-seq	HeLa	NA	NA	NA
human_m6A_14899	chr1:156018450	exonic	31257032	GSE122961	MAZTER-seq	HEK293T	NA	2	NA
human_m6A_14900	chr1:156023428	exonic	31257032	GSE122961	MAZTER-seq	HEK293T	NA	NA	NA

Abbrevations: m6A: N6-methyladenosine; UBQLN4: ubiquilin 4; GO: gene ontology; RBP: RNA binding protein; NA: not available.
