# Supplementary material for: A Comprehensive Multiomics Analysis Identified Ubiquilin 4 as a Promising Prognostic Biomarker of Immune-Related Therapy in Pan-Cancer
Source: J Oncol. 2021 Sep 7;2021:7404927. doi: 10.1155/2021/7404927 (PMC8443395; doi:10.1155/2021/7404927)
Supplement: Supplementary Materials — Supplementary Figure 1. The DSS analysis of UBQLN4 mRNA in pan-cancer based on TCGA database. The Kaplan–Meier curves of UBQLN4 in ACC (A), GBM (B), KICH (C), LGG (D), LIHC (E), MESO (F), PCPG (G), SARC (H), SKCM (I), and UVM (J) with significance based on TCGA database. (K) The cox regression analysis for DSS and UBQLN4 expression in 33 cancer types based on TCGA database. Abbreviations: DSS: disease-specific survival; UBQLN4: ubiquilin 4; TCGA: The Cancer Genome Atlas; ACC: adrenocortical carcinoma; BLCA: bladder urothelial carcinoma; BRCA: breast invasive carcinoma; CESC: cervical squamous cell carcinoma and endocervical adenocarcinoma; CHOL: cholangiocarcinoma; COAD: colon adenocarcinoma; DLBC: lymphoid neoplasm diffuse large B-cell lymphoma; ESCA: esophageal carcinoma; GBM: glioblastoma multiforme; HNSC: head and neck squamous cell carcinoma; KICH: kidney chromophobe; KIRC: kidney renal clear cell carcinoma; KIRP: kidney renal papillary cell carcinoma; LAML: acute myeloid leukemia; LGG: brain lower grade glioma; LIHC: liver hepatocellular carcinoma; LUAD: lung adenocarcinoma; LUSC: lung squamous cell carcinoma; MESO: mesothelioma; OV: ovarian serous cystadenocarcinoma; PAAD: pancreatic adenocarcinoma; PCPG: pheochromocytoma and paraganglioma; PRAD: prostate adenocarcinoma; READ: rectum adenocarcinoma; SARC: sarcoma; SKCM: skin cutaneous melanoma; STAD, stomach adenocarcinoma; TGCT: testicular germ cell tumor; THCA: thyroid carcinoma; THYM: thymoma; UCEC: uterine corpus endometrial carcinoma; UCS: uterine carcinosarcoma; UVM: uveal melanoma. Supplementary Figure 2. The DFI analysis of UBQLN4 mRNA in pan-cancer based on TCGA database. The Kaplan–Meier curves of UBQLN4 in READ (A), SARC (B), and UCEC (C) with significance based on TCGA database. (D) The cox regression analysis for DFI and UBQLN4 expression in 33 cancer types based on TCGA database. Abbreviations: DFI: disease-free interval; UBQLN4: ubiquilin 4; TCGA: The Cancer Genome Atlas; ACC: adrenocortical ca [file 7404927.f1.zip › 7404927.f1/Supplementary Table 6.pdf]

Supplementary Table 6. Detailed information of human_m6A_14897, including the RNA Binding Region, miRNA-Targets, predicted GO functions, and Co-methylated m6A sites.

RNA Binding Region for [Site: human_m6A_14897]

RNA binding protein	Database	Study	Binding Chromosome	Binding Start	Binding End
CSTF2	POSTAR2	GSE40859: GSM1003588	chr1	156006645	156006666
DDX55	POSTAR2	ENCODE	chr1	156006605	156006651
HNRNPC	POSTAR2	GSE56010: GSM1350199	chr1	156006615	156006657
NUDT21	POSTAR2	GSE37401: GSM917661	chr1	156006622	156006660
PCBP2	POSTAR2	ENCODE	chr1	156006640	156006719
PTBP1	POSTAR2	GSE57278: GSM1378377	chr1	156006600	156006680
YTHDF2	POSTAR2	GSE49339: GSM1197607	chr1	156006648	156006709

miRNA-Targets for [Site: human_m6A_14897]

miRNA Name	Target RNA	Target RNA Type	Method	Source	Start Position	End Position
hsa-miR-224	UBQLN4	mRNA	miRanda	miRanda	156006636	156006657
hsa-miR-370	UBQLN4	mRNA	miRanda	miRanda	156006643	156006663

Predicted GO functions of Site: human_m6A_14897;Gene:UBQLN4

GO Name	GO ID	P-Value
ascending aorta development	GO:0035905	0.00012
autophagy	GO:0006914	0.00064
process utilizing autophagic mechanism	GO:0061919	0.00095
macroautophagy	GO:0016236	0.00131
central nervous system neuron differentiation	GO:0021953	0.00171
lymphoid progenitor cell differentiation	GO:0002320	0.00177
aorta morphogenesis	GO:0035909	0.00177
regulation of phosphatidylinositol 3-kinase activity	GO:0043551	0.00215
columnar/cuboidal epithelial cell differentiation	GO:0002065	0.00239
establishment of mitochondrion localization, microtubule-mediated	GO:0034643	0.00257
establishment of mitochondrion localization	GO:0051654	0.00257
positive regulation of lipid kinase activity	GO:0090218	0.00257
intermediate filament cytoskeleton organization	GO:0045104	0.00302
positive regulation of JAK-STAT cascade	GO:0046427	0.00302
regulation of glial cell proliferation	GO:0060251	0.00351
regulation of lymphocyte apoptotic process	GO:0070228	0.00351
central nervous system development	GO:0007417	0.00397
ventral spinal cord development	GO:0021517	0.00404

cell differentiation in spinal cord	GO:0021515	0.0046
autonomic nervous system development	GO:0048483	0.0046
positive regulation of phospholipid metabolic process	GO:1903727	0.0046
positive regulation of neural precursor cell proliferation	GO:2000179	0.00519
placenta development	GO:0001890	0.00543
tyrosine phosphorylation of STAT protein	GO:0007260	0.00581
glial cell proliferation	GO:0014009	0.00581
aorta development	GO:0035904	0.00581
regulation of tyrosine phosphorylation of STAT protein	GO:0042509	0.00581
immune response-inhibiting signal transduction	GO:0002765	0.00645
immune response-inhibiting cell surface receptor signaling pathway	GO:0002767	0.00645
signal transduction downstream of smoothened	GO:0007227	0.00645
age-dependent general metabolic decline	GO:0007571	0.00645
N-terminal peptidyl-lysine acetylation	GO:0018076	0.00645
amino acid neurotransmitter reuptake	GO:0051933	0.00645
lymphatic endothelial cell differentiation	GO:0060836	0.00645
lymphatic endothelial cell fate commitment	GO:0060838	0.00645
stomach neuroendocrine cell differentiation	GO:0061102	0.00645
regulation of stomach neuroendocrine cell differentiation	GO:0061105	0.00645
oligodendrocyte progenitor proliferation	GO:0070444	0.00645
regulation of oligodendrocyte progenitor proliferation	GO:0070445	0.00645
mast cell proliferation	GO:0070662	0.00645
regulation of mast cell proliferation	GO:0070666	0.00645
kidney interstitial fibroblast differentiation	GO:0072071	0.00645
regulation of DNA-directed DNA polymerase activity	GO:1900262	0.00645
regulation of cell fate determination	GO:1905933	0.00645
regulation of pancreatic A cell differentiation	GO:2000226	0.00645
regulation of N-terminal peptidyl-lysine acetylation	GO:2000759	0.00645
regulation of forebrain neuron differentiation	GO:2000977	0.00645
mitochondrion localization	GO:0051646	0.00647
positive regulation of STAT cascade	GO:1904894	0.00647
cardiac chamber morphogenesis	GO:0003206	0.00658

Co-methylated m6A sites

Neighbor Site	Neighbor Gene	Gene Symbol
human_m6A_171029	7552	ZNF711
human_m6A_173519	7503	XIST
human_m6A_63391	115939	TSR3
human_m6A_159786	25987	TSKU
human_m6A_123185	85456	TNKS1BP1
human_m6A_65817	25851	TECPR1
human_m6A_55354	9338	TCEAL1
human_m6A_156806	51430	SUCO
human_m6A_96767	27148	STK36
human_m6A_42810	121665	SPPL3
human_m6A_137263	6659	SOX4
human_m6A_31655	23067	SETD1B
human_m6A_123163	54463	RETREG1
human_m6A_99045	5905	RANGAP1

human_m6A_158465	200576	PIKFYVE
human_m6A_175930	30849	PIK3R4
human_m6A_96485	54904	NSD3
human_m6A_55708	7026	NR2F2
human_m6A_159011	4741	NEFM
human_m6A_43434	9612	NCOR2
human_m6A_28709	23389	MED13L
human_m6A_163999	4067	LYN
human_m6A_38724	10128	LRPPRC
human_m6A_114554	158038	LINGO2
human_m6A_124128	3786	KCNQ3
human_m6A_141378	64771	ILRUN
human_m6A_13826	3321	IGSF3
human_m6A_43059	3091	HIF1A
human_m6A_120466	3280	HES1
human_m6A_168687	9950	GOLGA5
human_m6A_7001	11259	FILIP1L
human_m6A_48293	55215	FANCI
human_m6A_173734	149371	EXOC8
human_m6A_75062	100170841	EPOP
human_m6A_120467	131566	DCBLD2
human_m6A_147175	2055	CLN8
human_m6A_17311	54921	CHTF8
human_m6A_17747	9859	CEP170
human_m6A_162262	140459	ASB6

Abbrevations: m6A: N6-methyladenosine; UBQLN4: ubiquilin 4; GO: gene ontology.
